# Model-based global sensitivity analysis as applied to identification of anti-cancer drug targets and biomarkers of drug resistance in the ErbB2/3 network

**DOI:** 10.1016/j.ejps.2011.10.026

**Published:** 2012-07-16

**Authors:** Galina Lebedeva, Anatoly Sorokin, Dana Faratian, Peter Mullen, Alexey Goltsov, Simon P. Langdon, David J. Harrison, Igor Goryanin

**Affiliations:** a Centre for Systems Biology, University of Edinburgh, Edinburgh EH9 3JD, UK; b School of Informatics, University of Edinburgh, Edinburgh EH8 9AB, UK; c Breakthrough Research Unit and Division of Pathology, IGMM, University of Edinburgh, Western General Hospital, Edinburgh EH4 2XU, UK; d Okinawa Institute of Science and Technology, Tancha, Onna-son, Kunigami-gun, Okinawa 904-0495, Japan

**Keywords:** Global sensitivity analysis, ErbB network model, Anti-cancer drug targets, Biomarkers, Drug resistance, Combinatorial therapy

## Abstract

High levels of variability in cancer-related cellular signalling networks and a lack of parameter identifiability in large-scale network models hamper translation of the results of modelling studies into the process of anti-cancer drug development. Recently global sensitivity analysis (GSA) has been recognised as a useful technique, capable of addressing the uncertainty of the model parameters and generating valid predictions on parametric sensitivities.

Here we propose a novel implementation of model-based GSA specially designed to explore how multi-parametric network perturbations affect signal propagation through cancer-related networks. We use area-under-the-curve for time course of changes in phosphorylation of proteins as a characteristic for sensitivity analysis and rank network parameters with regard to their impact on the level of key cancer-related outputs, separating strong inhibitory from stimulatory effects. This allows interpretation of the results in terms which can incorporate the effects of potential anti-cancer drugs on targets and the associated biological markers of cancer. To illustrate the method we applied it to an ErbB signalling network model and explored the sensitivity profile of its key model readout, phosphorylated Akt, in the absence and presence of the ErbB2 inhibitor pertuzumab. The method successfully identified the parameters associated with elevation or suppression of Akt phosphorylation in the ErbB2/3 network. From analysis and comparison of the sensitivity profiles of pAkt in the absence and presence of targeted drugs we derived predictions of drug targets, cancer-related biomarkers and generated hypotheses for combinatorial therapy. Several key predictions have been confirmed in experiments using human ovarian carcinoma cell lines. We also compared GSA-derived predictions with the results of local sensitivity analysis and discuss the applicability of both methods. We propose that the developed GSA procedure can serve as a refining tool in combinatorial anti-cancer drug discovery.

## 1 Introduction

Development of prognostic and predictive models for diagnostics and therapeutic applications is one of the major goals of so-called mathematical oncology (Anderson and Quaranta, 2008; Auffray et al., 2009; Clermont et al., 2009). Network modelling techniques promise to substantially advance our understanding of the complexity of cancer-related pathways and likely mechanisms of disease (Chen et al., 2009; Hatakeyama, 2007; Kreeger and Lauffenburger, 2010; Nakakuki et al., 2008). However, examples of successful practical exploitation of pathway models to optimize anti-cancer therapies are rare. One case where a kinetic modelling approach has proved to be productive is in identifying novel anti-cancer drug targets (Schoeberl et al., 2009), based on the results of local sensitivity analysis. This led to the design of a novel drug candidate MM-121, which is a human monoclonal antibody that targets ErbB3 (Schoeberl et al., 2010). In our recent studies (Faratian et al., 2009b; Goltsov et al., 2011) we applied computational modelling methods, based on elucidation of control parameters within the PI3K/PTEN/Akt signalling module, to explore the mechanisms of therapeutic resistance to anti-ErbB2 inhibitors in human ovarian carcinoma cell lines. Thus we confirmed the role of quantitative PTEN protein expression as a key determinant and putative biomarker of therapeutic resistance.

One of the major barriers to more successful translation of the results of modelling studies into clinical practice and anti-cancer drug development is a high level of individual variability of the cellular networks involved in seemingly identical cancers, not only due to genomic abnormalities (Kan et al., 2010), but also complex post-transcriptional and post-translational variability in protein signalling networks (Faratian et al., 2009a). This causes a significant variation in individual responses to targeted anti-cancer treatments and therefore questions the practical utility of conclusions that can be drawn from network models with fixed parameters. Indeed, the majority of existing cancer-related modelling studies have been performed in a canonical way, where network model construction is followed by its parameterisation via fitting the model to experimental data, and further analysis of one or several best solutions (Birtwistle et al., 2007; Chen et al., 2009; Faratian et al., 2009b; Schoeberl et al., 2009). The experimental data, used for model calibration, usually represent a set of time-course profiles of changes in protein phosphorylation, observed in response to perturbation of signalling with various receptor ligands. Given that such data are normally registered for a particular cancer cell line, the quantitative predictions (e.g. on promising drug targets) drawn from the model analysis, though applicable to the reference cell type, may not be readily transferable to other subtypes of cancer, due to possible biological variation of the network parameters in different cell lines, as well as potential noise in parameter estimates caused by the noise in experimental data. This may explain the slow incorporation of systems biology approaches as credible clinical tools.

Another key but related impediment is the non-identifiability of model parameters, a problem common to many large-scale network models (Chen et al., 2009; Hengl et al., 2007; Rodriguez-Fernandez, 2006; Yue et al., 2006). In complex biochemical models many parameters remain uncertain even when additional data are generated and different fitting algorithms are implemented (Brown and Sethna, 2003; Chen et al., 2009). The majority of modelling studies employ various types of sensitivity analysis (SA) to assess how variation in input parameters can affect the model output. The most generally used method is local sensitivity analysis (LSA), based on evaluation of the impact of single parametric perturbations on the model output in close proximity to a reference solution, defined by nominal parameter values. From this basis, the predictions are made how oncogenic mutations may affect signal propagation through the network (Birtwistle et al., 2007; Chen et al., 2009) and thus potential targets for anti-cancer drugs are suggested (Schoeberl et al., 2009). Because of the poor identifiability of model parameters the reliability of the conclusions drawn from LSA remains a serious drawback.

Therefore there is a need to develop theoretical approaches capable of addressing individual variability of signalling networks, and drawing valid predictions from the models with uncertain parameters. One suitable framework, offering appropriate mathematical apparatus, is global sensitivity analysis (GSA). In contrast to LSA, which estimates the effect of small variations of individual parameters on the model output in a proximity to a single solution, GSA allows exploration of the sensitivity of model outputs to the simultaneous perturbation of multiple parameters within a parameter space (Marino et al., 2008; Saltelli, 2004; Saltelli et al., 2008; Zi et al., 2008). Recently there has been a growing recognition of the potential benefits of using GSA techniques for network model analysis (Balsa-Canto et al., 2010; Marino et al., 2008; Rodriguez-Fernandez and Banga, 2010). Although examples of the application of GSA to biochemical network models are still rare, they have already shown promise for understanding the effects of multi-parametric perturbations on biologically meaningful model outputs (Jia et al., 2007; Kim et al., 2010; Marino et al., 2008; Yoon and Deisboeck, 2009; Zheng and Rundell, 2006).

We propose a novel version of GSA, designed to explore the sensitivity of integrated model readouts to the perturbation of multiple model parameters within a parameter space, before and after a targeted anti-cancer drug is introduced into a network system. In our GSA implementation we place special emphasis on identifying a set of critical parameters, controlling the level of key output signals from the network, thereby providing a basis for generating hypotheses on potential anti-cancer drug targets, biomarkers of drug resistance, and combinatorial therapies. The predictions drawn from our method are based on the analysis and comparison of global sensitivity profiles of key model readouts in the absence and presence of the drugs.

We demonstrate the capabilities of our approach by applying it to our previously developed ErbB2/3 network model (Faratian et al., 2009b), exploring the sensitivity of its key model readout, pAkt, to simultaneous perturbation of all the model parameters in the absence and presence of the ErbB2 inhibitor pertuzumab. The GSA results, in addition to confirming our previous findings on the role of PTEN as one of the key biomarkers of resistance to anti-ErbB2 drugs, identified and allowed us to hypothesise that several additional network components (e.g. PDK1, PI3K, PP2A) significantly contribute to the control of network input–output behaviour. These components can be drug targets (e.g. PDK1, PI3K) or biomarkers of pertuzumab resistance (PI3K, PP2A), and have been confirmed by experimentation and recent findings. We also compare the results of GSA with LSA-derived predictions and discuss the applicability of each method.

## 2 Material and methods

### 2.1 ErbB2/3 network model

**Fig. 1:**
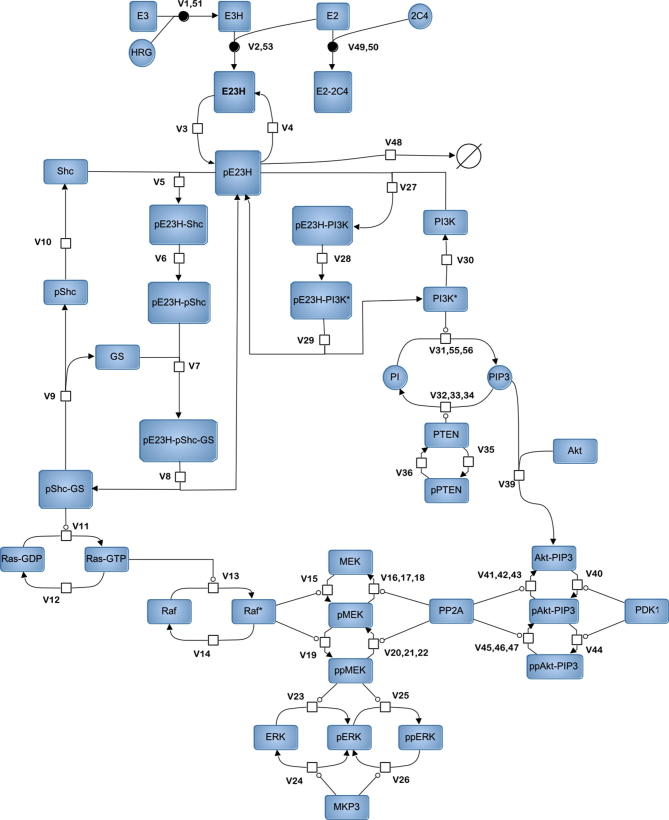
General scheme of the ErbB2/3 network model, presented in SBGN notation (Le Novere et al., 2009). Abbreviations used within the scheme are explained in Supplementary Table S1. Additional schemes for particular blocks of the model can be found in Additional File 1.

In (Faratian et al., 2009b) we developed a kinetic model of ErbB2/3 – related signalling in the PE04 human ovarian carcinoma cell line, and from it we predicted consequences of anti-ErbB2 monoclonal antibody therapeutic interventions. Here we briefly outline the model structure and highlight several minor modifications made for the purposes of this report. The general scheme for the model is shown in Fig. 1. The model includes the description of ErbB2 antibody receptor binding, ErbB2/ErbB3 dimerisation, Akt/MAPK signalling and crosstalk. It also includes a simplified mechanistic description of the PTEN catalytic cycle and Akt/MAPK crosstalk, via competition of phosphorylated forms of Akt and MEK for PP2A phosphatase and inhibition of active Raf by phosphorylated Akt.

In this contribution we introduced the following changes to our previously developed model:

(1) We neglected three reactions describing auto-dephosphorylation of PTEN (reactions 36–38 in previous model), and replaced them with a single generalized Michaelis-Menten-like reaction of PTEN dephosphorylation (reaction V36). This allowed us to significantly reduce the computation time, as recalculation of the balance between various PTEN forms for each parameter set no longer involved solving of an additional ODE subsystem as in the previous implementation. This gain in the performance was important due to the computationally intensive nature of GSA, which required running multiple simulations of the model.
(2) Two additional parameters (*k*_14_1_ and *K*_m,14_1_) were introduced into the reaction rate 14 describing deactivation of Raf by activated Akt, to separately account for the contribution of this feedback into the overall sensitivity of the system.

Additional schemes for the separate blocks of the model, corresponding ODE system and list of abbreviations are presented in Additional File 1, Supplementary Figs. S1–S4, and Supplementary Table S1. The modified model included 54 ODEs and 91 parameters; the SBML file of the model can be found in Additional File 4.

The resulting model was then recalibrated with the use of the same set of time-series data, as in (Faratian et al., 2009b), the time-course of protein phosphorylation in the PE04 ovarian carcinoma cell line after stimulation with heregulin in the presence and absence of the anti-ErbB2 inhibitor pertuzumab (see Fig. S6 in Additional File 1). The model was not fully identifiable. The results of identifiability analysis are presented in Additional File 1. The nominal parameter values, identified in one of the best fittings are presented in Additional File 2 and Supplementary Table S2.

### 2.2 General overview of the GSA method

While the general GSA theory has been under development for nearly three decades (Chang and Delleur, 1992; Saltelli et al., 1999), the potential of using GSA for systems biology applications has been recognised only relatively recently. Though the field is currently rapidly developing (Marino et al., 2008; Rodriguez-Fernandez and Banga, 2009; Rodriguez-Fernandez and Banga, 2010; Zi et al., 2008) there are no established standards in the choice of particular techniques for specific applications. Depending on the purpose of the analysis, various approaches have been suggested to incorporate GSA in the general pipeline of network model development and validation (Kim et al., 2010; Rodriguez-Fernandez and Banga, 2010; Zi et al., 2008).

In this study we sought to develop a GSA procedure which would be applicable to identification of the critical nodes that exhibit the most control over the output signals from cancer-related signalling networks, and therefore could be considered as candidates for targeting with anti-cancer drugs, or as biological markers of cancer and drug resistance.

Below we briefly outline the most popular GSA approaches currently in use, justify the choice of the techniques for our GSA procedure, describe the proposed algorithm and then highlight its applied aspects.

In general, all global SA techniques are designed to allow exploration of the model behaviour in the space of the model input factors. Therefore, at the first stage, they employ various sampling algorithms for extraction of parameter sets from predefined areas of parameter space. Then for each parameter set the model outputs are calculated, and various SA methods are applied to deduce particular metrics to quantitatively describe model input–output relationships. Thus, one way of classifying the existing GSA implementations would be to characterise them with regard to their choice of (1) the sampling method, (2) the method for sensitivity analysis, (3) the characteristic used to assess the parametric sensitivity.

#### 2.2.1 Choice of the sampling method

Classical “grid” approaches which would allow one to systematically cover the parameter space with “n” points on each individual parameter direction, cannot be used in a high-dimensional space, because of the exponential increase in volume associated with adding extra dimensions to a mathematical space that results in a computationally intractable task. That is why special sampling algorithms should be employed to effectively extract the points from a high-dimensional parameter space.

The most commonly used sampling methods are pure Monte-Carlo (MC), when points are taken randomly from multi-dimensional distribution (Balsa-Canto et al., 2010; Yoon and Deisboeck, 2009) and Latin Hypercube Sampling (LHS) (Jia et al., 2007; Marino et al., 2008). LHS, a variant of stratified sampling without replacement, ensures better estimation of the mean and the population distribution function compared to pure random MC sampling (Saltelli, 2004). In our GSA implementation, we used Sobol’s low-discrepancy sequence (LDS) as our sampling method (Sobol, 1998). Sobol’s LDS belongs to the class of quasi-random sampling methods, designed to systematically fill the gaps in the parameter space, rather than to select points purely randomly. LDS of quasi-random numbers has an important property in that the volume of any hypercube within parameter space, covered by LDS, is proportional to the number of sampled points within that hypercube. This allows LDS to cover the parameter space more evenly compared to MC and LHS. Each parameter combination, sampled by Sobol’s algorithm, is unique, which means that sampling of N Sobol’s points from a hypercube provides *N* variants of parameter value on each individual parameter direction.

#### 2.2.2 Choice of the SA method

Among the most popular methods of sensitivity analysis are averaged local sensitivities (Balsa-Canto et al., 2010; Kim et al., 2010; Zi et al., 2008), Sobol’s method (Kim et al., 2010; Rodriguez-Fernandez and Banga, 2010; Zi et al., 2008), Partial Rank Correlation Coefficient (PRCC) (Marino et al., 2008; Zi et al., 2008), and Multi-Parametric Sensitivity Analysis (MPSA) (Yoon and Deisboeck, 2009; Zi et al., 2008). In general, different SA methods are better suited to specific types of analysis. For example, analysis of a distribution of local sensitivities, can be very useful for the initial scoring of parameters prior to model calibration, especially if sensitivity coefficients can be derived analytically and will not require numerical differentiation, which significantly increases the computational cost.

The choice of the particular SA method significantly depends on the assumed relationship between the input parameters and model output. If a linear trend can be assumed, the methods based on calculation of the Pearson correlation coefficient can be employed. For nonlinear but monotonic dependences, PRCC and standardized rank regression coefficient (SRRC) appear to be the best choice (Marino et al., 2008), as they work with rank transformed values. If no assumption can be made about the relationship between model inputs and outputs, or the dependence is non-monotonic, another group of sensitivity methods can be employed, based on decomposition of the variance of the model output into partial variances, assessing the contribution of each parameter to the total variance. One of the most powerful variance-based methods is Sobol’s method; however it is also known to be among the most computationally intensive, with the cost growing exponentially with the dimensionality of the parameter space (Rodriguez-Fernandez and Banga, 2010). Another promising method that makes no assumptions about the dependence between model parameters and outputs is MPSA (Jia et al., 2007; Yoon and Deisboeck, 2009). In MPSA all outputs are divided into two groups: “acceptable” and “unacceptable” and parameter distributions in both groups are tested against the null hypothesis that they are taken from the same distribution. The lower is the probability of acceptance of null hypothesis, the higher is the sensitivity of the parameter (Zi et al., 2008). When binary decomposition of model outputs can be naturally introduced the results of MPSA can be very useful (Yoon and Deisboeck, 2009).

In our GSA implementation we chose to use PRCC as the preferred method for SA, as one of the most efficient and reliable sampling-based techniques (Marino et al., 2008). Importantly, PRCC provides the sign of the sensitivity index for each parameter, thereby allowing interpretation of sensitivity profiles in terms of inhibitions/activations of corresponding proteins, which suits well the purpose of our analysis. One caveat of the method is that it presumes a monotonic dependence of the model output on the input parameters, which may not always be true. In case of unknown or non-monotonic dependence MPSA could be a better choice. Importantly, during the testing of the method on the ErbB2/3 network model, the preliminary visual analysis of the scatterplots revealed no significant non-monotonicity in the relationship between input parameters and key model outputs (see Additional File 3). This justified the choice of PRCC in this particular case.

#### 2.2.3 Choice of the characteristic for the analysis

The choice of the characteristic for sensitivity analysis is key to the method and depends on the specific purpose of the analysis. The majority of known GSA implementations have been designed to support the model calibration process. Therefore their natural choice was to analyse the metrics derived from the distance between a reference solution, defined by nominal parameters (or experimental data) and a set of new solutions, defined by the sampled parameter sets.

**Fig. 2:**
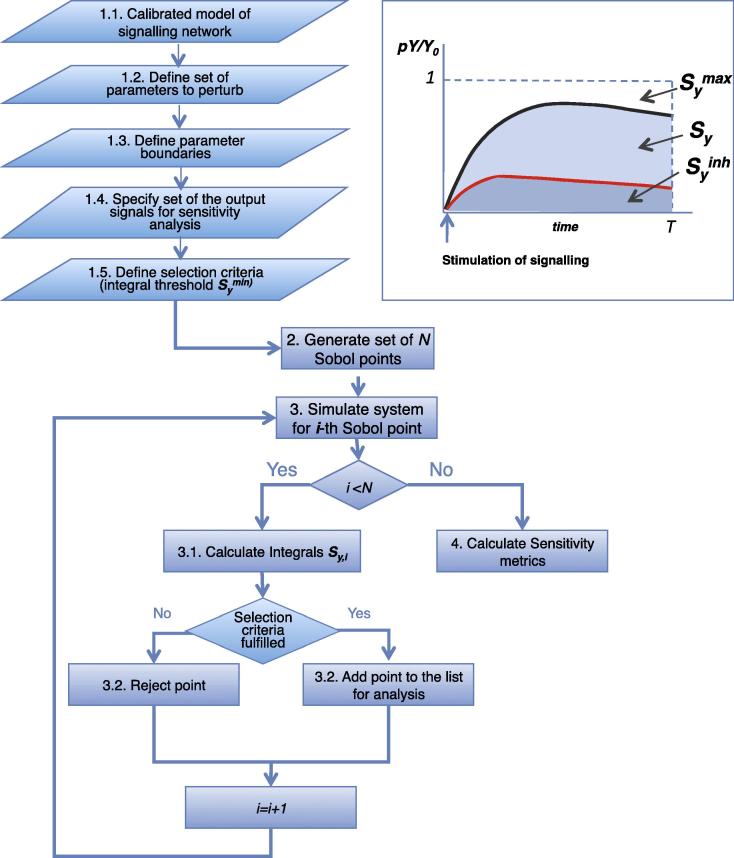
Overview of method. Key stages of GSA applied to integrated model readouts. Inset: Scheme explaining the characteristic used for sensitivity analysis. *S_y_* and $S_{y}^{\mathrm{Inh}}$ – area under the normalised time-course profile of phosphorylated protein *Y* in the absence or presence of the inhibitor *Inh*, $S_{y}^{\max}$ – theoretical maximal value of *S_y_*.

In developing our method, we pursued another goal: to employ GSA techniques for identification of anti-cancer drug targets and biomarkers within signalling networks. Therefore our GSA procedure should be capable of answering biologically-relevant questions, namely, which components of signalling networks have the dominant control over the value of key signal outputs, when the majority of network parameters are uncertain. For this reason, in our procedure we focussed on the analysis of a biologically-relevant characteristic – the area under the time-course profile (*S_y_*) of the phosphorylated states of key signalling proteins (see Fig. 2, inset), which can be computed as definite integrals of the corresponding model species.

The use of such a characteristic has certain benefits. Firstly, the characteristic conveys a sense of the total exposure of the cellular microenvironment to the signal, represented by an activated signalling protein, over a given period of time, and therefore allows us to study the overall effectiveness of signal processing at the level of each protein. Secondly, *S_y_* of the key signalling components can be directly related to the particular cellular response to stimulation, such as proliferation or survival. For example, as shown in (Asthagiri et al., 2000) the integrated ERK2 activity was proportional to DNA synthesis, and therefore could be used as a quantitative measure of cell proliferation. Finally, analysis of *S_y_* allowed us to overcome problems associated with individual variability of time-course profiles, such as transient dips, peaks, possible oscillations, slower/faster kinetic profiles, etc. In our approach such variations do not contribute to the resulting sensitivity other than by changing the overall area under the curve.

Similar attempts to use biologically meaningful characteristics in GSA procedure have been presented in Yoon and Deisboeck (2009) and Kim et al. (2010). Yoon et al. used MPSA to identify network components controlling Erk responses to be either transient or sustained. For this purpose, two characteristic measures were introduced, the amplitude and the duration of the Erk signal, to split all parameter sets into binary classes. In Kim et al. Sobol’s algorithm was applied to predict the parameters that control the characteristic, related to the delay time to cell death – a biologically-relevant quantity, which was not a state variable of the model.

In both studies application of GSA techniques provided a valuable insight into the mechanism controlling input–output behaviour of the networks, with potential to be used for identification of biomarkers for pharmaceutical drug discovery processes.

### 2.3 GSA implementation: description of the algorithm

The flowchart of our GSA procedure is presented in Fig. 2. Further we briefly outline key stages of the proposed GSA procedure and illustrate how each of them was implemented for our test system – ErbB2/3 network model.

**Step 1: Definition of the inputs to the method**

In our GSA implementation the inputs to the method include:

S.1.1. *A kinetic model of a signalling pathway, calibrated on a set of time-series data*

Because of our specific interest in identification of anti-cancer drug targets and the analysis of drug resistance, our version of GSA uses as an input a kinetic model of a signalling pathway, calibrated on a particular set of time-series data. Any model calibrated in this way should contain a set of parameters, identified from a fitting procedure, to achieve the best match between experimental curves and relevant model trajectories. Suitable data represent time course profiles of phosphorylated proteins, registered after stimulation of the signalling with relevant receptor ligands. Our ErbB2/3 network model was calibrated on the set of time course profiles of pErbB3, pErk and pAkt registered after stimulation of PE04 cells with heregulin, in the presence and absence of anti-ErbB2 inhibitor pertuzumab (see (Faratian et al., 2009b) and Fig. S6 in Additional File 1).

Note that in general GSA does not require a calibrated model as an input, but here calibration is needed to confirm the validity of the model. However, full identifiability of the model is not required.

S.1.2. *Definition of a set of model parameters to perturb*

Depending on the purpose of the analysis the set can include either all system parameters or a particular sub-set.

In our analysis of the ErbB2/3 network we perturbed all model parameters, including kinetic constants and total concentrations of the signalling proteins, with exception of the parameters corresponding to the concentration of external compounds, such as receptor ligands (heregulin-β, (HRG)) and inhibitors (pertuzumab (Per)), which were fixed at their values used in the experiments.

S.1.3. *Definition of the parameter boundaries for GSA*

Setting the boundaries of the parameter space for GSA for large scale models represents a distinct task, as on the one hand, they should be relatively wide to justify the globality of the analysis, but on the other hand the boundaries should be reasonably narrow due to the limitations imposed by the resulting computational time and available CPU resources. Since our GSA implementation is specifically directed towards identification of appropriate drug targets and cancer-related biomarkers within signalling networks, the parameter ranges should be able to incorporate potential effects of drugs and genetic modifications on the level of protein activities. In our analysis we assumed that up to a 10-fold reduction in parameter value could imitate an efficient suppression of the protein activity by an anti-cancer drug. It’s worth noting, that it is difficult to predict the real extent of the inhibition of the protein activity by targeted drugs *in vivo*, since it depends on many factors – drug transformations within the body, efficiency of drug delivery to the target, etc. However, there is a good reason to believe that *in vivo* drugs cause not more than a 10-fold inhibition of targeted protein activity. For example, in our experiments pertuzumab caused up to 40% inhibition of ErbB3/2 dimer formation (Faratian et al., 2009b). Recent findings of Gaborit et al. (2011) also confirmed that anti-ErbB2 drugs cause not more than 40–20% of reduction of ErbB2 heterodimerization, when used alone, and up to 70%, when combined with an EGFR inhibitor. These estimates have been made for drugs targeting cellular membrane receptors. For intracellular targets the level of inhibition may be even lower, due to additional factors, limiting drug availability within the cell (e.g. due to inefficient drug transfer into the cell).

Similarly, we assumed that up to a 10-fold variation of parameter value above and below its nominal value (that in total provides effectively a 100-fold variation) could approximate modification of protein activity by the majority of mutations. For example, a PIK3CA mutation is thought to increase PI3K activity only two-fold (Carson et al., 2008), whereas lipid phosphatase activity of PTEN can differ up to 100-fold between different PTEN mutants, as assessed in (Rodriguez-Escudero et al., 2011). Importantly, in our analysis the parameters are varied *within* the 10-fold range around the nominal value, thus allowing us to consider many possible levels of protein inhibition/activation, including both weak and strong effects.

Thus, for our ErbB2/3 network model the constraints for the majority of kinetic parameters were set to span one order of magnitude above and below the values obtained in one of our best data fits. In some cases the parameter ranges were adjusted to match the order of magnitude of other existing estimates (see Additional File 2 and Table S2). For most of proteins the total concentration was varied between 10 and 1000 nM, since the majority of existing estimates for components of ErbB network fall within this range (Birtwistle et al., 2007; Hatakeyama et al., 2003; Kholodenko et al., 1999; Klinke, 2010).

S.1.4. *Definition of the model readouts subject to sensitivity analysis*. At this stage the model readouts for inclusion in the analysis should be specified. In principal, GSA can be applied to any number of model outputs or combination of them, but in practice it is sensible to focus on the analysis of one or several most informative model readouts.

For the ErbB2/3 network model we explored the output signal from the PI3K/Akt branch of the network, focusing on the analysis of the time course profile of phosphorylated Akt (pAkt), where pAkt was defined as the composition of several model species, corresponding to different forms of phosphorylated Akt, normalised by the total concentration of Akt protein:

pAkt=([pAkt-PIP3]+[ppAkt-PIP3]+[pAkt-PIP3-PP2A]+[ppAkt-PIP3-PP2A])/Akt_tot

S.1.5. *Definition of the criteria to include/reject a parameter set into/from the analysis*. Quasi-random parameter sets sampled from the parameter space correspond to a variety of system behaviours, some of them potentially biologically implausible. Depending on the purpose of the analysis, at this stage the criteria for classifying parameter sets as plausible/implausible should be formulated. For the ErbB2/3 network model, we included in the analysis only those parameter sets, for which the phosphorylation level of Akt in the absence of the drug exceeded 1% of the total Akt protein.

**Step 2: Sampling N parameter sets from the hypercube**

To sample the points from the hypercube defined by parameter ranges we use Sobol’s LDS algorithm, which ensures that individual parameter ranges are evenly covered (Joe and Kuo, 2003; Sobol, 1998), implementation taken from (http://people.sc.fsu.edu/~burkardt/cpp_src/sobol/sobol.html).

The choice of the adequate sample size (*N*) depends on the properties of the system. One way to estimate the optimal *N* is to systematically increase the sample size and check, whether the set of the most sensitive parameters keeps changing with the increase of *N*. When two consecutive experiments consistently capture and rank a similar set of most important parameters, one can conclude that there is no obvious advantage in further increasing the sample size.

For our ErbB2/3 network model we used a quantitative metric “top-down coefficient of concordance” (TDCC) to assess the adequacy of the sample size *N*, as suggested by Marino et al. (2008). TDCC is a measure of correlation between parameter ranks found in two consecutive sampling experiments, which is designed to be more sensitive to agreement on the top rankings (Iman and Conover, 1987). We calculated TDCC for sample size *N* = [5000, 10,000, 30,000, 40,000, 50,000, 80,000, 100,000, 120,000]. Starting from *N* = 50,000 TDCC followed a saturation trend, reaching the value of 1 at *N* = 100,000 (see Supplementary Fig. S9 in Additional File 3). Thus we estimated 120,000 as a sufficient number of Sobol’s points for our analysis.

**Step 3: Simulating the system for each parameter set and classifying solutions**

S.3.1. *Calculating integral metrics for sensitivity analysis*

For each randomly selected parameter set (Sobol point) we run a simulation of the model and then calculate the area under the time course profiles of the model readouts of interest (see inset to Fig. 2):

$$S_{y}=\int_{0}^{T}y(t)\mathrm{dt}$$

where $y=\frac{\mathrm{pY}}{Y_{0}}$ stands for the concentration of the phosphorylated form *pY* of the protein *Y* (for instance, pErk, pAkt), normalised to the total concentration of the given protein (*Y*_0_), *T* – time span for integration.

In our further analysis we used a normalised dimensionless version of this metric:

$$S_{y\text{,}n}=S_{y}/S_{y}^{\max}\text{,}$$

where $S_{y}^{\max}$ is a theoretical maximal value of *S_y_*, which could be achieved if all the protein *Y* were phosphorylated in a sustained manner. Thus *S_y_*_,_*_n_* varies in the range from 0 to 1 and represents the actual fraction of the potential maximal signal, produced by protein *Y*. Therefore *S_y_*_,_*_n_* can be interpreted as the relative effectiveness of signal generation at a given signalling stage.

The choice of the adequate time span for integration T is dictated by the characteristic time of system response to perturbation, which should be experimentally confirmed. In our GSA implementation we set T in such a way to fully capture transient dynamics of changes in protein phosphorylation observed in response to stimulation of the signalling with receptor ligands. For the ErbB2/3 network system our experiments confirmed that *T* = 60 min was a sufficient period of time for the key signalling components (e.g. pAkt, pErk) to fully develop the response to stimulation of the signalling with heregulin (see Additional File 1 and Fig. S6).

Thus, for the ErbB2/3 network model, for each parameter set we ran two simulations imitating two typical settings used in the experimental study: stimulation of ErbB2/3 signalling with heregulin-β (1) in the absence and (2) in the presence of anti-ErbB2 inhibitor, pertuzumab, and calculated the area under the 60 min pAkt time course profile: *S_pAkt_* and $S_{\mathrm{pAkt}}^{\mathrm{Per}}$. Both metrics were normalised by $S_{\mathrm{pAkt}}^{\max}$.

S.3.2. *Classifying calculated metrics S_y,n_ as acceptable/unacceptable for further analysis*

This has been done in accordance with selection criteria defined at stage 1.5. Parameter sets for which *S_pAkt_*_,_*_n_* < 0.01 has been excluded from the analysis.

**Step 4. Calculating sensitivity indices for key model readouts**

To analyse the sensitivity of the integral characteristics *S_y_* to the variation of model parameters we use a variant of Partial Rank Correlation Coefficient (PRCC) analysis (Saltelli, 2004; Zheng and Rundell, 2006), implemented in R package ‘sensitivity’. The calculated PRCC sensitivity indices are a standardized sensitivity measure representing correlation between the value of model readout *S_y_*_,_*_n_* and model parameter *P_j_* with removed influence of the correlation of parameter of interest with other parameters. To reduce the influence of nonlinearity, the correlation is calculated based upon ranks rather than absolute values.

PRCC between *P_j_* and *S_y,n_* was calculated as the correlation coefficient $r_{p_{j}s}$ between the two residuals $p_{j}={\overset{ˆ}{P}}_{j}-{\overset{˜}{P}}_{j}$ and $s={\overset{ˆ}{S}}_{y\text{,}n}-{\overset{˜}{S}}_{y\text{,}n}$, where ${\overset{ˆ}{P}}_{j}$ and ${\overset{ˆ}{S}}_{y\text{,}n}$ are rank transformed *P_j_* and *S_y,n_*; ${\overset{˜}{P}}_{j}$ and ${\overset{˜}{S}}_{y\text{,}n}$ are the linear regression models defined as follows (Marino et al., 2008):

P˜j=a0+∑l=1l≠jkalPˆl;S˜y,n=b0+∑l=1l≠jkblPˆl

Thus

$$r_{p_{j}s}=\frac{\sum_{i=1}^{N}(p_{\mathrm{ij}}-\overset{¯}{p})(s_{i}-\overset{¯}{s})}{\sqrt{\sum_{i=1}^{N}{\left(p_{\mathrm{ij}}-\overset{¯}{p}\right)}^{2}\sum_{i=1}^{N}{\left(s_{i}-\overset{¯}{s}\right)}^{2}}}\text{,}$$

where *N* is the number of Sobol’s points sampled from the model parameter space; $\overset{¯}{p}$ and $\overset{¯}{s}$ are respective sample means.

Importantly, the sign of a PRCC indicates how the variation of each parameter affects the output signal: the positive index corresponds to the parameter whose higher value is likely to be associated with a higher value of the model output, and vice versa. The value of PRCC indices are distributed between – 1 and 1 with 0 indicating an input to which the model output is completely insensitive.

**Fig. 3:**
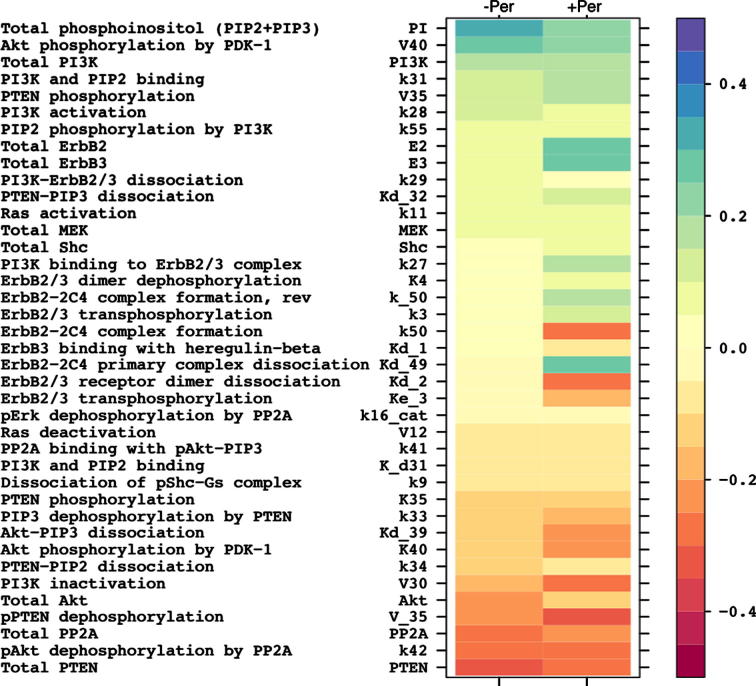
GSA applied to the prediction of drug targets and biomarkers in ErbB2/3 network. The sensitivity spectrum of the integrated pAkt model readout to the simultaneous perturbation of all kinetic parameters and total concentrations of proteins, in the absence (left) and presence (right) of pertuzumab. The 40 most sensitive parameters are shown. The values of sensitivity coefficients are colour-coded according to the scale shown on the right. All sensitivity coefficients with absolute value greater than 0.05 are significant at the confidence level of 95%. The full sensitivity spectrum for *S_pAkt_* and $S_{\mathrm{pAkt}}^{\mathrm{Per}}$ is presented in Additional File 3, Supplementary Fig. S10.

Thus, the output from our GSA procedure represents a matrix of PRCC, which contains the quantitative metrics of how the variation of each model parameter is correlated to the value of the integrated model readouts (*S_y_*_,_*_n_*) of interest. To facilitate the analysis of the matrix, the results are visualised in the form of colour-coded sensitivity profiles for individual model readouts *S_y_*_,_*_n_*. For the ErbB2/3 network model we generated the sensitivity profiles for *S_pAkt_* and $S_{\mathrm{pAkt}}^{\mathrm{Per}}$ (see Fig. 3).

### 2.4 Applied aspects of the GSA method: interpretation of GSA profiles

The main goal of targeted anti-cancer treatments is to inhibit particular components within signalling networks in order to suppress signal propagation through the particular branches that have been recognised as implicated in cancer progression.

Our GSA methodology has been designed for identification of the network parameters whose variation has the most impact on the value of the key signalling network outputs. Therefore we propose, that it can be used for the prediction of potential drug targets and biomarkers of cancer and drug resistance.

Such predictions can be derived from the analysis and comparison of the sensitivity profiles of key model readouts in the absence (*S_y_*) and in the presence ($S_{y}^{\mathrm{Inh}}$) of the targeted drugs (inhibitors). In particular, we assume that the *S_y_* sensitivity profile can be used to identify anti-cancer drug targets and biomarkers of susceptibility to cancer, as it points to the parameters, variation of which is most likely to be associated with the suppression or elevation of cancer-related model outputs *S_y_*. At the same time, the analysis of $S_{y}^{\mathrm{Inh}}$ may help to predict potential biomarkers of drug resistance and generate ideas of suitable combination therapies, as it identifies the parameters for which the model readout retains sensitivity after the drug has been introduced. An illustration of practical application of the method to the ErbB2/3 network model is given in Section 3.

### 2.5 Local sensitivity analysis

To create local sensitivity spectrum of our model parameters, each nominal parameter *P_i_* was incremented and decremented by 1% of its value (*dp_i_*) and the normalised sensitivity coefficient for the area under the pAkt time course profile was calculated as follows (Zi et al., 2008):

$$C_{i}^{\mathrm{pAkt}}=\frac{\frac{S_{\mathrm{pAkt}}(P_{i}+{\mathrm{dP}}_{i})-S_{\mathrm{pAkt}}(P_{i}-{\mathrm{dP}}_{i})}{S_{\mathrm{pAkt}}(P_{i})}}{\frac{2{\mathrm{dP}}_{i}}{P_{i}}}$$

### 2.6 Computations

The construction and calibration of the ErbB2/3 model was carried out with the use of the DBsolve package for kinetic modelling (Gizzatkulov et al., 2010; Goryanin et al., 1999). All GSA-related computations were run on Edinburgh University ECDF cluster: 10 nodes were used to run simulations of ODE system for 120,000 Sobol’s points; 200 nodes were used to calculate PRCC indexes for sensitivity analysis. Thus an average analysis took 20 h for model simulation and two hours for sensitivity analysis. ODE system was solved using CVODE solver from SUNDIALS package (Hindmarsh et al., 2005), sensitivity analysis was performed with the package ‘sensitivity’ (http://cran.r-project.org/web/packages/sensitivity/index.html) in R environment (http://www.r-project.org/).

### 2.7 Experimental methods

#### 2.7.1 Cell culture and treatment of cells

PE04 and OVCAR4 cells were grown as monolayer cultures in RPMI supplemented with 10% heat-inactivated foetal calf serum (FCS) and penicillin/streptomycin (100 IU/mL) in a humidified atmosphere of 5% CO_2_ at 37 °C. Time course experiments were set up by plating cells into 10 cm diameter petri dishes and leaving for 24 h. Cells were then briefly washed in PBS before transferring to phenol red-free DMEM containing 5% double charcoal-stripped serum supplemented with penicillin/streptomycin (100 IU/mL) and glutamine (0.3 mg/mL) for a further 48 h prior to treatment. Cells were treated with UCN-01 (protein kinase inhibitor; Calbiochem #539644; final concentration of 1 μM), LY294002 (PI3 kinase inhibitor; Calbiochem #440204; final concentration 20 μM), Pertuzumab (ErbB2 inhibitor; final concentration 100 nM) and stimulation by Heregulin (R&D Systems; 396-HB-CF) was at final concentration of 1 nM. Cells were treated for 15 min with the aforementioned drugs as appropriate immediately followed by the addition of heregulin-β (1 nM). The concentrations of drugs used in the experiments corresponded to the dose causing 50% inhibition of cell growth.

#### 2.7.2 Collection of lysates and western blot analysis

Samples were collected at time points of 1, 5, 30, and 60 min after initiation of heregulin treatment, washed in PBS, and immediately lysed in ice-cold isotonic lysis buffer [50 mM Tris–HCl (pH 7.5), 5 mM EGTA (pH 8.5), 150 mM NaCl, 1% Triton X-100] supplemented with aprotinin (10 μg/mL), phosphatase inhibitor cocktail A (Sigma, P2850), phosphatase inhibitor cocktail B (Sigma, P5726) and a protease inhibitor cocktail (Roche, 11836153001). Lysates were centrifuged for 6 min at 13,000*g* and protein concentrations of supernatants subsequently determined using the BCA assay (Sigma, BCA-1).

#### 2.7.3 In cell western blotting

PE04 and OVCAR4 cells were plated out in black 96-well round bottom trays and left for 24 h after which they were washed in PBS and transferred to 5% double charcoal-stripped serum-containing media as described above. Following drug treatment, media was aspirated and cells were fixed in 100 μl of 4% formaldehyde for 15 min at room temperature before being washed three times in PBS. Cells were permeabilized with 100 μl of ice-cold methanol for 10 min at −20 °C and again washed in PBS. Staining was performed by blocking for 60 min at room temperature (5% goat serum/0.3% Triton X-100 in PBS) after which primary antibody incubations (in 1% BSA/0.3% Triton X-100 in PBS) were carried out overnight at 4 °C. Antibodies to pAKT (Cell Signalling; #9271 at 1:50) and total AKT (Cell Signalling; #2920 at 1:50, were optimized for InCell western incubations. Secondary antibody detection was carried out as described for western blot analysis with 1:800 IRdye^680^ (for the normalizer) and 1:800 of IRdye^800^ (for the target). Analysis was carried out after pAKT: tAKT normalisation.

#### 2.7.4 Reverse phase protein arrays (RPPA)

Denatured and reduced protein lysates were spotted onto nitrocellulose-coated glass slides (Whatman, Stamford, ME) using a MicroGrid II robotic spotter (DigiLab, Holliston, MA) as previously described (Spurrier et al., 2008). Three replicates were spotted per sample in five two-fold dilutions (resulting in a total of 15 spots per sample). Slides were hydrated in Li-Cor blocking buffer for 1 h (LI-COR Biosciences, Nebraska, USA), and then incubated with primary antibodies overnight at 4 °C in a sealed box containing a damp paper towel. Antibodies to pAKT (Cell Signalling; #9271 at 1:50), and PP2A (Cell Signalling; #2259 at 1:50), were optimized for RPPA incubations. Slides were stained using matched total and phospho-proteins duplexed on each slide. The following day slides were washed three times in PBS/0.1% Tween 20 (PBS-T) at room temperature for 5 min before incubating with far-red fluorescently-labelled secondary antibodies diluted in Li-Cor Blocking Buffer (1:2000) at room temperature for 45 min with gentle shaking. Slides were then washed in excess PBS/T (x3)/PBS (x3) and allowed to air dry before reading on a Li-Cor Odyssey scanner at 680 nm and 780 nm. RPPA analysis was performed using MicroVigene RPPA analysis module (VigeneTech, Carlisle, MA, USA). Spots were quantified by accurate single segmentation, with actual spot signal boundaries determined by the image analysis algorithm. Each spot was quantified by measuring the total pixel intensity of the area of each spot (volume of spot signal pixels), with background subtraction of 2 pixels around each individual spot. The quantification y0 (intensity of curve) or rsu (relative concentration value) of sample dilution curves were normalised using the corresponding total protein.

## 3 Results and discussion

### 3.1 Testing the GSA procedure: application to ErbB2/3 network model

Though our GSA procedure is suitable for sensitivity analysis of any number of model readouts, in this study, for demonstration purposes, we focused on the analysis of a single output from the ErbB2/3 model – the timecourse of Akt phosphorylation. This has been done for a number of reasons. Firstly, the elevated pAkt signalling has been implicated as a major determinant of cancer (Faratian et al., 2009b; Schoeberl et al., 2009); secondly, the level of Akt phosphorylation has been indicated as the key responsive element to anti-ErbB2 inhibitors and to the changes in ErbB2 expression (Birtwistle et al., 2007; Faratian et al., 2009b).

Below we present the results of the analysis of the *S_pAkt_* global sensitivity profile in the presence and absence of ErbB2 inhibitor pertuzumab, and demonstrate what useful information can be drawn from the analysis.

#### 3.1.1 Analysis of pAkt global sensitivity profile helps to identify potential drug targets and biomarkers of susceptibility to cancer

The *S_pAkt_* sensitivity spectrum (Fig. 3, left column) can be interpreted in the following way: lower values of the parameters, shown at the top of the spectrum, in general correspond to a lower pAkt signal, while lower values of the parameters at the bottom of the diagram are likely to result in a higher value of *S_pAkt_*, and vice versa.

Thus the parameters at both poles of the spectrum would point to the proteins whose activity, if dysregulated (via activating mutations or activity loss), could result in elevated pAkt signalling. Therefore these proteins could serve as biomarkers of dysregulated PI3K/Akt signalling in cancer. The parameters from the upper part of the spectrum would indicate promising drug targets, as their lower values would correspond to lower *S_pAkt_*, and therefore targeting these proteins may be beneficial with respect to suppressing pAkt.

In the absence of the drug (Fig. 3) the pAkt signal had most of its sensitivity concentrated on the parameters related to the function of the PI3K/PTEN/Akt signalling branch, whereas the sensitivity to the majority of parameters of the MAPK branch was in a near zero range. Similar lack of sensitivity of the pAkt signal to the parameters of MAPK cascade has been previously reported in (Schoeberl et al., 2009).

The highest sensitivity (positive correlation) of *S_pAkt_* was found for the parameters describing the size of the phosphoinositol pool (PI), the maximal rate of Akt phosphorylation by PDK1 (V40), and several other parameters of PI3K/PTEN signalling cycle. The total amount of PTEN and PP2A, as well as several parameters related to their catalytic activity were negatively correlated with the value of the pAkt signal.

Thus, our GSA procedure identified the phosphoinositol pool (PI), PDK1 and PI3K as the most promising targets to suppress *S_pAkt_*. At the same time, hyper-activation of PDK1 and/or PI3K, as well as the loss of PTEN and/or PP2A activity, were highlighted as potential biomarkers of Akt pathway dysregulation in cancer.

We next sought the confirmation of these predictions in experiments and from the available literature. The direct manipulation of PI pool is not advisable for drug therapy, due to intricate involvement of multiple PI derivatives in many important physiological processes, including contraction of cardiomyocytes. Instead, pool of phosphoinositols can be targeted indirectly, via inhibiting the proteins (e.g. PI3K), controlling the balance between various PI forms. Therefore we focused on testing the effect of PI3K and PDK1 inhibition on the level of Akt phosphorylation in two ovarian carcinoma cell lines, PE04 and OVCAR4. These two cell lines were chosen for the following reasons: PE04 was used as a reference cell line for initial model calibration; OVCAR4 was chosen because it had an expression profile, in general, similar to PE04 for the key Erk/Akt pathway proteins (ErbB1-3, PTEN, PI3K, Akt, Erk (see Faratian et al., 2009b), but had a noticeably different response to pertuzumab. For example, in growth inhibition studies OVCAR4 demonstrated a high level of resistance to pertuzumab, in contrast to PE04, which was pertuzumab responsive. A low level of expression of ErbB1 receptors in both cell lines allowed us to assume that the general structure of our ErbB2/3 network model was suitable for describing HRG-induced signalling in both cell lines. The observed discrepancy in the PE04 and OVCAR4 response to pertuzumab thus could be attributed to the differences in the corresponding network parameters, that made OVCAR4 a suitable candidate for testing the GSA predictions. Indeed, our GSA procedure was designed to allow extension of the predictions generated with the use of the model, calibrated for a particular cell line (PE04), to other cell lines with the same network topology (in our case OVCAR4), without the need to fit the model to any new data sets.

**Fig. 4:**
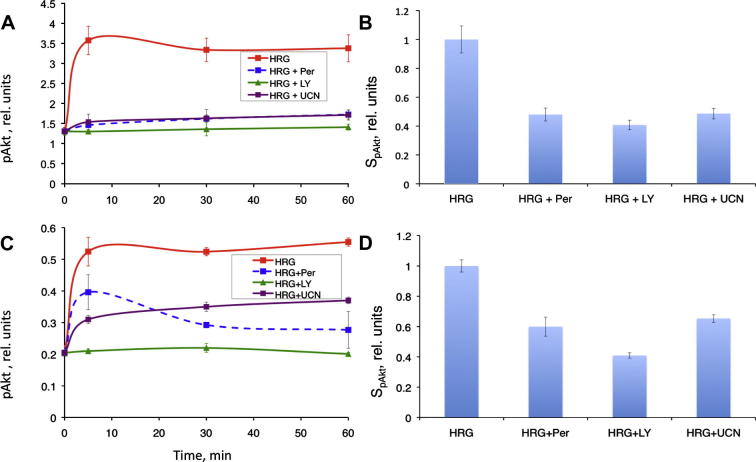
Testing GSA predictions of drug targets in ErbB2/3 network. Experimental confirmation of drug targets, predicted by GSA, in PE04 (A and B) and OVCAR4 (C and D) cell lines: time course profile (left) and integrated pAkt response (right) to heregulin-β stimulation ± pertuzumab (Per), LY294002 (LY) and UCN-01 (UCN). All integrals were normalised on the value of *S_pAkt_* observed in the absence of any inhibitors. The error-bars indicate 95% confidence intervals of technical replicates.

We stimulated the PE04 and OVCAR4 cells with heregulin after pre-treating them either with LY294002 (PI3K inhibitor) or UCN-01 (PDK1 inhibitor). To compare the resulting inhibitory effect with the efficiency of the existing drugs, we also measured the effect of pertuzumab on Akt phosphorylation, as this ErbB2 inhibitor is currently in clinical trials for the therapy of breast and ovarian cancer. Both tested compounds effectively inhibited the pAkt signal in both cell lines (Fig. 4), however the effect of UCN-01 was more pronounced in the PE04 cell line, than in OVCAR4, which may result from a higher Akt expression in OVCAR4 as compared to PE04 (Faratian et al., 2009b). In both cell lines LY294002 demonstrated higher than pertuzumab potency in suppressing the pAkt signal, whereas the effect of UCN-01 was comparable to that of pertuzumab.

Our findings with regard to PI3K and PDK1 as potential drug targets and biomarkers of cancer are consistent with other cancer-related studies (Iorns et al., 2009; Peifer and Alessi, 2009). Both PDK1 and PI3K are currently attractive lead targets in clinical trials. Overstimulation of PDK1 has been found in >50% of all human cancers (Peifer and Alessi, 2008), including ovarian cancer (Ahmed et al., 2008). PI3K pathway activation is a frequent event in ovarian cancer (Kan et al., 2010), and clinical trials are underway using PI3K inhibitors (Coughlin et al., 2010).

Our theoretical findings with regard to PTEN and PP2A as potential biomarkers of elevated pAkt, are in agreement with current understanding of their role in the onset and progression of cancer. Mutations causing dysregulation of PTEN activity has been implicated in a number of human cancers (Blanco-Aparicio et al., 2007; Tamguney and Stokoe, 2007). The role of PP2A in controlling the level of pAkt has been confirmed by Perrotti and Neviani (2008), who observed that inhibition of PP2A was associated with sustained phosphorylation of proteins, whereas re-activation of PP2A led to cell growth suppression.

#### 3.1.2 Analysis of the pAkt sensitivity profile after drug administration allows identification of biomarkers of drug resistance and potential combination therapy

One of the key assumptions underlying our approach is that the introduction of a drug modifies the properties of the biochemical network, including its sensitivity to parameter variation, and that analysis of such modifications can help to tackle the mechanisms of drug resistance.

Indeed, the sensitivity spectrum of the integrated pAkt signal after pertuzumab administration (Fig. 3, right column), though retaining most of the sensitivity found in the absence of the drug, exhibited a number of significant differences (see Additional File 3 for detailed analysis and discussion of changes). The additional parameters for which pAkt acquired higher sensitivity in the presence of the drug were mainly related to the “upstream” component of the signalling pathway, corresponding to signal propagation through the level of receptors.

From the analysis of the $S_{\mathrm{pAkt}}^{\mathrm{Per}}$ sensitivity profile we identified potential biomarkers of pertuzumab-resistance and targets for combination therapy. In particular, the parameters negatively correlated with $S_{\mathrm{pAkt}}^{\mathrm{Per}}$ were considered biomarkers of pertuzumab resistance, since lower values of these parameters, or loss of activity of corresponding proteins, were associated with higher values of $S_{\mathrm{pAkt}}^{\mathrm{Per}}$. Conversely, the proteins whose activity was positively correlated with $S_{\mathrm{pAkt}}^{\mathrm{Per}}$ were considered as potential targets for combination therapy with pertuzumab.

*Biomarkers of resistance to pertuzumab*. The analysis of the $S_{\mathrm{pAkt}}^{\mathrm{Per}}$ sensitivity profile confirmed our previous findings that the loss of PTEN activity is a key biomarker of resistance to pertuzumab (Faratian et al., 2009b). Indeed, compared to *S_pAkt_*, $S_{\mathrm{pAkt}}^{\mathrm{Per}}$ (Fig. 3) remained sensitive to the level of PTEN, and acquired even higher sensitivity to the parameters of the PTEN–phospho-PTEN turnover. Other parameters negatively correlated to $S_{\mathrm{pAkt}}^{\mathrm{Per}}$ were related to PP2A, indicating that loss of PP2A activity also may be considered a biomarker of pertuzumab resistance. We tested this in a panel of 12 ovarian carcinoma cell lines (Faratian et al., 2009b), and the quantitative expression of PP2A was positively correlated with growth inhibition by pertuzumab (Spearman’s Rank Correlation 0.434; Supplementary Fig. S11 in Additional File 3). Similarly, $S_{\mathrm{pAkt}}^{\mathrm{Per}}$ became more sensitive to the parameters controlling PI3K activity, which is in agreement with experimental findings implicating PI3K activation mutations in resistance to anti-ErbB drugs (Blanco-Aparicio et al., 2007; Coughlin et al., 2010).

*Predictions on drug combinations*. The highest sensitivity of $S_{\mathrm{pAkt}}^{\mathrm{Per}}$ was found for the total amount of ErbB3 and ErbB2, which confirms that expression level of these receptors plays a significant role in modulating the response of the ErbB network to anti-ErbB2 inhibitors. In (Schoeberl et al., 2009) ErbB3 was identified as a key node in controlling pAkt, which led directly to the design of a novel anti-ErbB3 inhibitor MM-121. According to our analysis, simultaneous inhibition of both ErbB3 and ErbB2 by a combination of drugs might result in a greater suppression of pAkt, as compared to mono-therapy with an ErbB2 inhibitor (not tested).

**Fig. 5:**
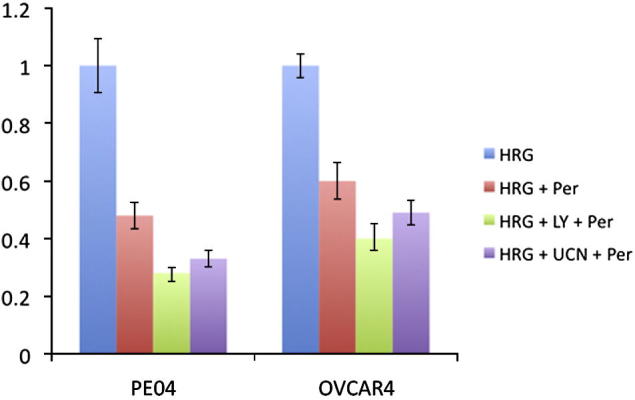
Experimental testing of GSA-derived combination therapies. Integrated pAkt response to heregulin-β stimulation in PE04 (left) and OVCAR4 (right) cell lines, treated with combinations of pertuzumab, LY294002 (LY) and UCN-01 (UCN). The data were normalised on the AUC of pAkt time-course in the absence of any drugs.

Importantly, in the presence of the drug, $S_{\mathrm{pAkt}}^{\mathrm{Per}}$ retained relatively high sensitivity to the parameters of PI3K and PDK1, which indicates that the compounds, targeting these proteins, could be candidates for combination therapy with pertuzumab. We tested this by measuring the effect of LY294002 and UCN-01 combined with pertuzumab in the PE04 and OVCAR4 cell lines. Both drug combinations were effective, showing additional inhibition of pAkt as compared to pertuzumab alone (Fig. 5).

### 3.2 Comparison with local sensitivity analysis (LSA)

The majority of existing cancer-related modelling studies employ local sensitivity analysis methods (LSA) to assess the impact of single parametric perturbations on the model readouts of interest. Based on this, conclusions are drawn on the potential inhibitory or stimulatory effects of oncogenic mutations on the level of the network output signals (Birtwistle et al., 2007; Chen et al., 2009) and predictions of potential targets for anti-cancer therapies are generated (Schoeberl et al., 2009). However, LSA has some serious limitations which should be taken into consideration when interpreting local sensitivity metrics in terms related to drug discovery. Firstly, in traditional LSA methods the parameters are varied only in a localised region around the nominal parameter values, and sensitivity metrics are derived under the assumption that there is a linear relationship between input parameters and model outputs. At the same time drug effects presume significant suppression of the targeted protein activity, which can result in non-linear system responses. Secondly, in LSA implementations only a single parameter is perturbed at a time, while the rest of parameters remain fixed at their values identified from the best fitting. In cancer cells the network parameters may be subjected to significant biological variation. These limitations, along with the poor identifiability of the parameters in the large-scale network models, raise questions about the possibility of extending LSA-derived conclusions to more general cases of highly variable networks and large parametric perturbations.

In this context, GSA approach has important advantages. Indeed, in contrast to LSA, GSA evaluates the effects of large-scale parameter perturbation on model outputs, that allows imitation of strong inhibitory or activation effects caused by modern targeted therapeutics or oncogenic mutations. GSA is also more flexible with regard to assumptions about the relationships between input parameters and analysed model outputs. It can effectively work either with no assumption about the nature of this relationship (e.g. variance-based GSA methods) or with an assumption about monotonicity of such dependence (e.g. PRCC, used in our implementation). Moreover, random sampling of parameter space, employed by GSA, may imitate biological variability of network parameters in different cells and cell lines, caused by genetic variations and post-translational modifications. Importantly, our GSA implementation can make use of poorly identifiable models, that, in contrast to LSA, makes our method even less dependent on the nominal parameter values, identified in fitting.

In this study we performed the comparison of LSA and GSA-derived predictions, using our reference ErbB2/3 network model as a test system. For this purpose we ran local sensitivity analysis of the ErbB2/3 model in the proximity of the best solution, identified from fitting. To make LSA results more comparable with GSA findings, in our LSA implementation we used the same characteristic (area under pAkt time course profile) for sensitivity analysis (see Methods for details).

**Fig. 6:**
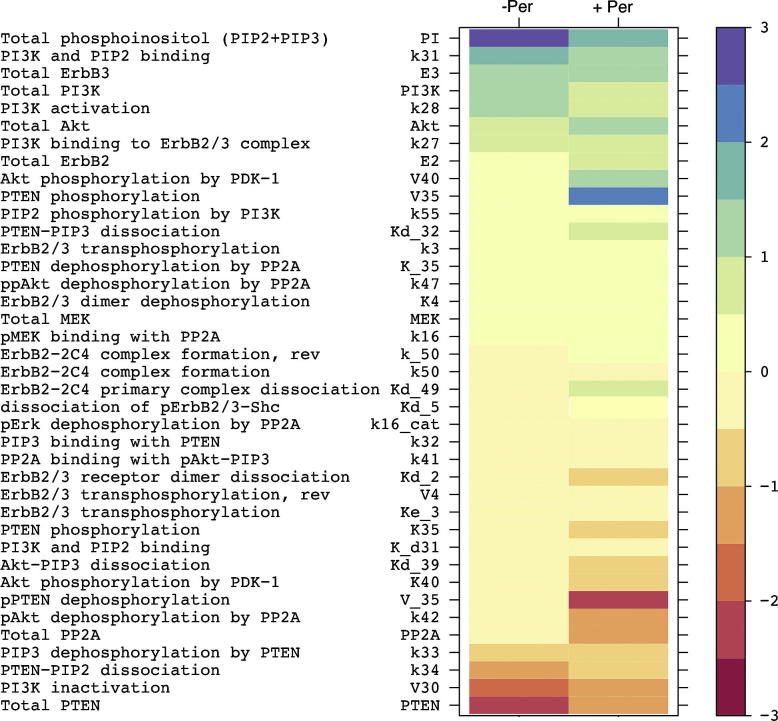
Local sensitivity analysis of ErbB2/3 network model. The sensitivity of the integrated pAkt time-course profile to the single-parametric perturbation of kinetic parameters and total concentrations of proteins, calculated in the proximity to the reference solution for the absence (left) and presence (right) of pertuzumab (Per).

As can be seen from comparison of Fig. 3 and Fig. 6, most sensitive parameters identified by LSA were also present in GSA-derived sensitivity spectrum, but there were some noticeable discrepancies in the rank of parameters obtained by local and global sensitivity methods. Similarly to GSA, in the absence of pertuzumab, LSA indicated highest sensitivity for the total amount of phosphoinositol (PI) and PTEN. High sensitivity was also confirmed for the parameters of PI3K/PTEN signalling cycle (k28, k31,k34, total PI3K). However, LSA indicated ErbB3 as one of the key parameters controlling the level of pAkt phosphorylation, whereas in GSA ErbB3 had a significantly lower rank. Moreover, while GSA predicted high sensitivity for the rate of Akt phosphorylation by PDK1 (V40), in LSA V40 was positioned much lower in the spectrum. Interestingly, in Schoeberl et al. (2009) (Schoeberl et al., 2009) LSA also revealed ErbB3 as the key node in controlling pAkt, whereas, in contrast to our findings, the sensitivity for the parameters of PI3K and PDK1 was found to be very low.

Similarly, commonalities and differences can be found in the LSA and GSA profiles generated in the presence of pertuzumab (Fig. 6, right column): LSA predicted the most sensitivity for the parameters of PTEN-phospho-PTEN turnover (V35 and V_35), while the sensitivity to total PTEN and PI3K dropped compared to the “no pertuzumab” case. GSA, along with confirming PTEN as a key node in the control of pAkt signal in the presence of pertuzumab, also predicted a noticeable increase in the sensitivity for the parameters of the receptor module: total ErbB2,3, receptor–ligand and receptor–dimer complex formation. Both methods indicated PDK1 as a sensitive node in the presence of pertuzumab. GSA predicted higher sensitivity to PI3K than LSA.

To summarise, most of the parameters identified by LSA in this study represented a subset of GSA derived predictions, but the LSA ranking differed from the GSA ranking. Such differences in the predictions provided by global and local sensitivity methods, as well as the discrepancy between LSA findings presented in different studies, in our opinion, should not be considered as contradictory, because they originate from significantly different design and purposes behind local and global types of analysis.

Indeed, LSA is normally performed in the proximity of the single solution identified from the best fitting to a particular dataset, therefore it would be logical to expect that it can help to identify the proteins possessing the most control over the output signal in the particular cell line used for model calibration. For example, LSA of our ErbB2/3 network model could point to the best targets to suppress the pAkt signal in the PE04 ovarian carcinoma cell line. However, since the model is not fully identifiable, such predictions may not be accurate. In contrast to LSA, GSA works not with a single model solution, but with the whole ensemble of those, generated for N randomly sampled parameter sets. Therefore GSA procedure is not intended to find the best targets for inhibition in a particular cell type, but instead it identifies those proteins whose parameters are highly correlated with the output signal of interest in the majority of (but not all) possible network implementations, defined by possible combinations of network parameters. Thus, the GSA of our ErbB2/3 network model points to the proteins, targeting of which is likely to result in a lower pAkt signal in the majority of cells with the same network topology, while the kinetic parameters of individual reactions may differ between the cells or be uncertain.

Because of the differences in technical setup and applicability of LSA and GSA techniques, we suggest that these methods should not be opposed but rather considered as complementary approaches, which, when used together, may allow exploration of a wider range of promising targets and prioritisation for future study. Indeed our GSA procedure predicted that PDK1 could be a promising target to suppress pAkt. In contrast to that conclusion, LSA indicated a very low level of sensitivity to PDK1, both in our study and in Schoeberl et al. (2009) (Schoeberl et al., 2009). Experimental testing of GSA prediction proved that inhibition of PDK1 resulted in a significant suppression of pAkt signal in two cell lines, including PE04, which was used for initial calibration of our model. In addition to this, GSA identified loss of PP2A activity as a potential biomarker of elevated pAkt, while in LSA the sensitivity of pAkt to PP2A parameters was very low. Thus GSA helped to predict an additional potential drug target (PDK1) and a putative biomarker (PP2A), which have not been captured by LSA. At the same time, in contrast to LSA findings, our GSA has not indicated ErbB3 as a promising target in the absence of ErbB2 inhibitors, whereas targeting ErbB3 was shown to effectively suppress pAkt signalling in ADRr and OvCAR8 cancer cell lines (Schoeberl et al., 2009).

## 4 Conclusions

Systems biology is advancing only very slowly in actually making a contribution to cancer research. There is a tension between the individual variability and the uncertainty of the parameters of biochemical networks involved in cancer onset and progression, which hamper the translation of the results of network modelling studies into anti-cancer drug development. Moreover, a potentially significant level of network perturbations caused by anti-cancer drugs or oncogenic mutations questions the applicability of local sensitivity analysis for anti-cancer drug development, since LSA works with small-scale parameter perturbations. This emphasises the need for development of theoretical approaches and methods capable of addressing the uncertainty of model parameters and generating valid predictions about the behaviour of critical network outputs under large-scale multi-parametric perturbations.

In this study we investigated and confirmed the value of global sensitivity analysis as a powerful technique for the analysis of network models with uncertain parameters, which shows good promise for practical applications in anti-cancer drug discovery. We present a novel implementation of model-based GSA, intended for identification of drug targets and biological markers within cancer-related signalling networks. Our GSA procedure is based on Sobol’s LDS sampling method and employs PRCC to perform the sensitivity analysis. Importantly, in our procedure we focus on the sensitivity analysis of a biologically meaningful characteristic – the area under the time-course profile of phosphorylated proteins, that allows us to assess the effect of multi-parametric variations on the value of key cancer-related network outputs (e.g. phosphorylated Akt). Since PRCC provides the sign for the sensitivity indexes, our GSA implementation allows separation of strong negative and positive effects of parametric variations, thus facilitating interpretation of the resulting sensitivity profiles in terms of inhibition or activation of corresponding protein activities. The applied aspects of the method are based on the analysis and comparison of GSA profiles of cancer-related model outputs in the absence and presence of the drug.

As an illustrative example, we applied our method to a modification of our previously developed model of the ErbB2/3 signalling network (Faratian et al., 2009b) with a view to predict potential drug targets, drug combinations, and biomarkers of resistance to the anti-ErbB2 inhibitor pertuzumab. Some of the key predictions were tested experimentally in ovarian carcinoma cell lines. Our GSA procedure indicated PDK1 and PI3K as promising targets to suppress Akt phosphorylation, suggesting that the efficient suppression of pAkt signal can be achieved both with single drugs (a PDK1 or a PI3K inhibitor), and with combinations of each of these compounds with anti-ErbB2 inhibitor pertuzumab. Our experiments confirmed that both the PDK1 inhibitor UCN-01, and the PI3K inhibitor LY294002, effectively inhibited pAkt signalling in two different ovarian carcinoma cell lines, when used as single drugs and in combination with pertuzumab. Our findings with regard to potential biomarkers of pertuzumab resistance (PTEN, PP2A, PI3K) were in agreement with our own data (Faratian et al., 2009b; Goltsov et al., 2011) and other existing studies.

Importantly, many of the targets and biomarkers identified by our GSA procedure have been previously highlighted in other experimental and modelling studies, that can be considered as a confirmation of the predictive capabilities of the method.

Since LSA method still remains the most popular way for deriving quantitative predictions from ODE-based models, in this contribution we focussed on the discussion of our GSA procedure in comparison with this popular technique. We argue that GSA can substantially add value to the analysis of cancer-related network models, since, in contrast to LSA, it can successfully deal with the poor identifiability and uncertainty of the parameters associated with such models.

The comparison of the GSA and LSA predictions, generated for our reference ErbB2/3 network system, revealed that control parameters, highlighted by LSA represented a subset of GSA-derived predictions; importantly, these two methods assigned significantly different ranks to some of the key network parameters (e.g. ErbB3, PDK1, PP2A). We suggest that the observed discrepancy in LSA and GSA predictions may originate from substantial differences in theoretical assumptions and technical implementation of these methods, that define their range of applicability. LSA may be suitable to identify critical network components within particular cell type, used for initial model calibration, whereas GSA can help to explore a wider range of possible targets, which are likely to be valid for the majority (but not all) possible network implementations.

Though we have illustrated our GSA procedure on a single relatively well known system of ErbB associated signalling, we suggest that the proposed method may have broader applicability, since the general pipeline of our procedure is based on well-established and tested statistical and computational techniques. However, for the method to produce meaningful results, the input network model should satisfy certain criteria. Firstly, since our method works with integrated model trajectories, the model should be calibrated on a suitable perturbation time-course data and match experimentally observed system responses to stimulation, such as changes in protein phosphorylation after addition of receptor ligands. Secondly, because of the choice of PRCC analysis as the core method of sensitivity analysis, our current GSA implementation presumes monotonicity of relationship between model parameters and analysed network outputs. Therefore, prior to analysis, the tests should be made, whether such an assumption can be justified (e.g. via visual evaluation of relevant scatterplots). If the monotonicity of input–output relationship cannot be assumed, the GSA procedure would require further adjustments, including replacement of PRCC analysis with a more appropriate method of SA (e.g. MPSA).

## 5 Authors’ contributions

GL conceived the idea of the study, contributed to GSA design and coordination of the study, ran simulations, analysed and interpreted GSA and LSA results and wrote the manuscript. AS contributed to design of the study, implemented and ran GSA and LSA procedure, participated in interpretation of results and drafting the manuscript. DF, SPL, DJH planned the experiments, analysed data, contributed to drafting the manuscript. AG contributed to ErbB2/3 model development. PM performed the RPPA and in cell Western studies. SPL, DJH and IG contributed to design and coordination of the study, gave valuable advice and critically revised the manuscript. All authors read and approved the final manuscript.
