# Supplementary material for: Model-based global sensitivity analysis as applied to identification of anti-cancer drug targets and biomarkers of drug resistance in the ErbB2/3 network
Source: Eur J Pharm Sci. 2012 Jul 16;46(4):244–58. doi: 10.1016/j.ejps.2011.10.026 (PMC3398788; doi:10.1016/j.ejps.2011.10.026)
Supplement: Supplementary data 1 [file mmc1.doc]

**Additional File 1**

**Supplementary information on ErbB2/ErbB3 network model**

**Table of contents**

Supplementary Figures S1-S4: Additional kinetic schemes for separate blocks of ErbB2/3 network model [2](#__RefHeading___Toc169594018)

System of ordinary differential equations [5](#__RefHeading___Toc169594019)

Supplementary Table S1. List of species used in the ErbB2/3 network model and their abbreviations in the text, diagrams and SBML file [11](#__RefHeading___Toc169594020)

Identifiability analysis of ErbB2/ErbB3 network model [13](#__RefHeading___Toc169594021)

Supplementary Figure S5. Non-identifiable parameters of ErbB2/3 network model. [14](#__RefHeading___Toc169594022)

Supplementary Figure S6: ErbB2/3 model calibration [15](#__RefHeading___Toc169594023)

### Supplementary Figures S1-S4: Additional kinetic schemes for separate blocks of ErbB2/3 network model


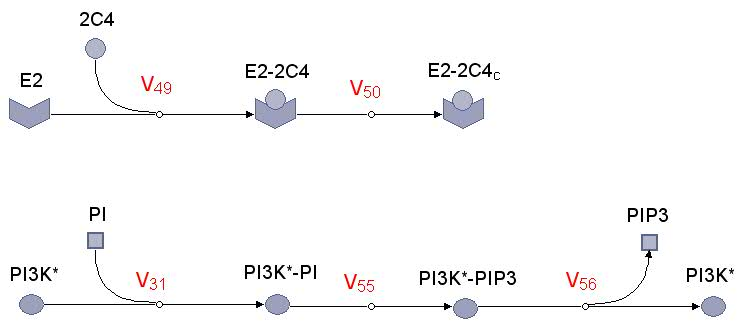


a

b

**Supplementary Figure S1.** Detailed schemes for the processes considered in the model: (a) the two-step mechanism of binding of the ErbB2 receptor (E2) with ligand, pertuzumab (2C4); (b) the reaction of PI phosphorylation catalysed by PI3K*.


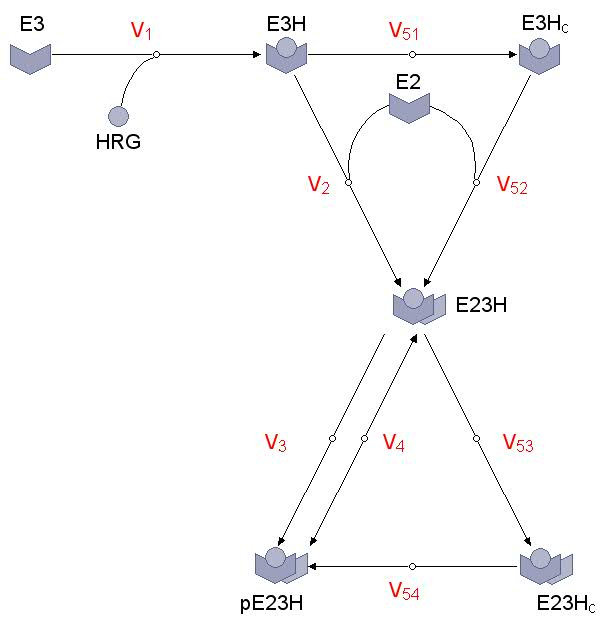


**Supplementary Figure S2.** Detailed scheme of heterodimerisation of ErbB3 (E3) and ErbB2 (E2) receptors followed by formation of transphosphorylated complex, pE23H. Reactions V_1_, V_51_ describe the two-step mechanism of ErbB3 binding with heregulin-β (HRG), through the formation of the intermediate, E3H, and the final, E3H_c_ , ligand-receptor complexes. Reactions V_2_, V_52_, and V_53_ represent a two-step mechanism of heterodimerisation of ErbB3 and ErbB2 receptors leading to formation of the intermediate E23H and the final E23H_c_ heterodimers. Reactions V_3_ and V_54_  describe transphosphorylation of E23H and E23H_c_ heterodimers with formation of pE23H complex. V_4_ – reaction of pE23H dephosphorylation.


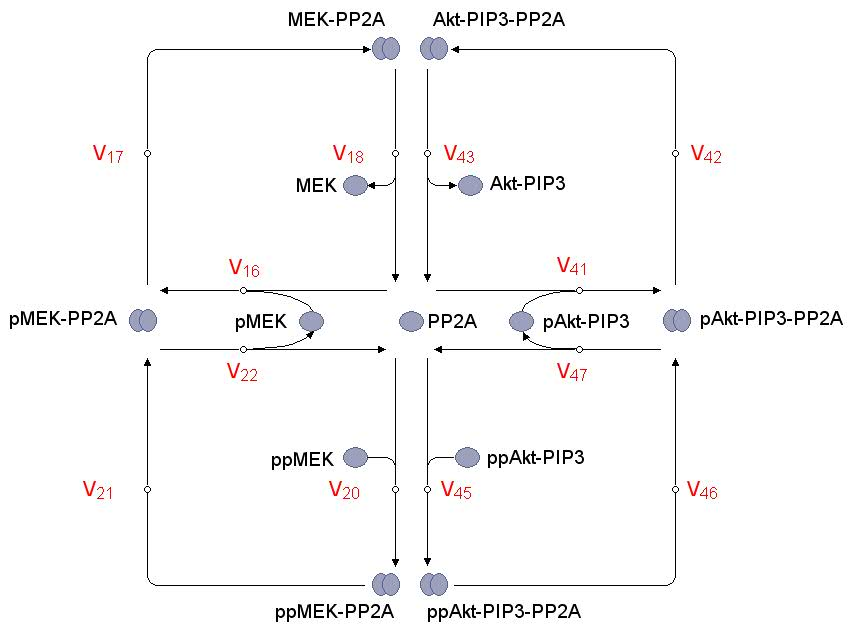


**Supplementary Figure S3.** Detailed scheme of dephosphorylation of pMEK (reactions V_16_, V_17_, V_18_) , ppMEK (reactions V_20_, V_21_, V_22_), pAkt (reactions V_41_, V_42_, V_43_), ppAkt (reactions V45, V46, V47) by phosphatase PP2A.


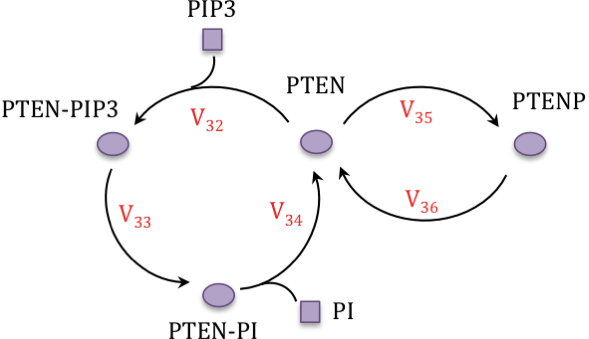


**Supplementary Figure S4.** Scheme of the PTEN catalytic cycle (reactions V_32_, V_33_, V_34_ , describing PTEN lipid phosphatase activity) and phosphorylation -dephosphorylation of PTEN (reactions V_35_, V_36_).

### System of ordinary differential equations

$\frac{d[\text{E3]}}{dt}=\text{-V}_{1}$ (S1.1)

$\frac{d[\text{E3H]}}{dt}=\text{V}_{1}\text{-V}_{2}-\text{V}_{51}$ (S1.2)

$\frac{d[\text{HRG]}}{dt}=\text{-V}_{1}$ (S1.3)

$\frac{\text{d[E3H}_{\text{c}}\text{]}}{\text{dt}}\text{=V}_{51}\text{-V}_{52}$ (S1.4)

$\frac{d[\text{E2]}}{dt}=\text{-V}_{2}\text{-V}_{49}\text{-V}_{52}$ (S1.5)

$\frac{d[\text{E23H]}}{dt}=\text{V}_{2}\text{-V}_{3}\text{+V}_{4}\text{+V}_{52}\text{-V}_{53}$ (S1.6)

$\frac{d[\text{E23H}_{\text{c}}]}{dt}=\text{V}_{53}\text{-V}_{54}$ (S1.7)

$\frac{d[\text{pE23H]}}{dt}=\text{V}_{3}\text{-V}_{4}\text{-V}_{5}\text{+V}_{8}\text{-V}_{27}\text{+V}_{29}\text{-V}_{48}\text{+V}_{54}$ (S1.8)

$\frac{d[\text{Shc]}}{dt}\text{=-V}_{5}\text{+V}_{10}$ (S1.9)

$\frac{d[\text{pE23H-Shc]}}{dt}=\text{V}_{5}\text{-V}_{6}$ (S1.10)

$\frac{d[\text{pE23H-pShc]}}{dt}=\text{V}_{6}\text{-V}_{7}$ (S1.11)

$\frac{d[\text{GS]}}{dt}=\text{-V}_{7}\text{+V}_{9}$ (S1.12)

$\frac{d[\text{Shc-GS]}}{dt}=\text{V}_{8}\text{-V}_{9}$ (S1.13)

$\frac{d[\text{pShc]}}{dt}=\text{V}_{9}\text{-V}_{10}$ (S1.14)

$\frac{d[\text{pE23H-pShc-GS]}}{dt}=\text{V}_{7}\text{-V}_{8}$ (S1.15)

$\frac{d[\text{ppAkt-PIP3]}}{dt}=\text{V}_{44}\text{-V}_{45}$ (S1.16)

$\frac{d[\text{Ras-GDP]}}{dt}=\text{-V}_{11}\text{+V}_{12}$ (S1.17)

$\frac{d[\text{Ras-GTP]}}{dt}=\text{V}_{11}\text{-V}_{12}$ (S1.18)

$\frac{d[\text{Raf]}}{dt}=\text{-V}_{13}\text{+V}_{14}$ (S1.19)

$\frac{d[\text{Raf*]}}{dt}=\text{V}_{13}\text{-V}_{14}$ (S1.20)

$\frac{d[\text{MEK]}}{dt}=\text{-V}_{15}\text{+V}_{18}$ (S1.21)

$\frac{d[\text{pMEK]}}{dt}=\text{V}_{15}\text{-V}_{16}\text{-V}_{19}\text{+V}_{22}$ (S1.22)

$\frac{d[\text{PP2A]}}{dt}=\text{-V}_{16}\text{+V}_{18}\text{-V}_{20}\text{+V}_{22}\text{-V}_{41}\text{+V}_{43}\text{-V}_{45}\text{+V}_{47}$ (S1.23)

$\frac{d[\text{MEKP-PP2A]}}{dt}=\text{V}_{16}\text{-V}_{17}\text{+V}_{21}\text{-V}_{22}$ (S1.24)

$\frac{d[\text{MEK-PP2A]}}{dt}=\text{V}_{17}\text{-V}_{18}$ (S1.25)

$\frac{d[\text{pAkt-PIP3-PP2A]}}{dt}=\text{V}_{41}\text{-V}_{42}\text{+V}_{46}\text{-V}_{47}$ (S1.26)

$\frac{d[\text{ppMEKPP-PP2A]}}{dt}=\text{V}_{20}\text{-V}_{21}$ (S1.27)

$\frac{d[\text{ppMEKPP-PP2A]}}{dt}=\text{V}_{20}\text{-V}_{21}$ (S1.28)

$\frac{d[\text{ppMEK]}}{dt}=\text{V}_{19}\text{-V}_{20}$ (S1.29)

$\frac{d[\text{ERK]}}{dt}=\text{-V}_{23}\text{+V}_{24}$ (S1.30)

$\frac{d[\text{pERKP]}}{dt}=\text{V}_{23}\text{-V}_{24}\text{-V}_{25}\text{+V}_{26}$ (S1.31)

$\frac{d[\text{ppERK]}}{dt}=\text{V}_{25}\text{-V}_{26}$ (S1.32)

$\frac{d[\text{ppAkt-PIP3-PP2A]}}{dt}=\text{V}_{45}\text{-V}_{46}$ (S1.33)

$\frac{d[\text{Akt-PIP3]}}{dt}=\text{V}_{39}\text{-V}_{40}\text{+V}_{43}$ (S1.34)

$\frac{d[\text{PI3K]}}{dt}=-\text{V}_{27}\text{+V}_{30}-\text{V}_{58}$ (S1.35)

$\frac{d[\text{pE23H-PI3K]}}{dt}=\text{V}_{27}\text{-V}_{28}$ (S1.36)

$\frac{d[\text{pE23H-PI3K*]}}{dt}=\text{V}_{28}\text{-V}_{29}$ (S1.37)

$\frac{d[\text{PI3K*]}}{dt}=\text{V}_{29}\text{-V}_{30}\text{-V}_{31}\text{+V}_{56}$ (S1.38)

$\frac{d[\text{PI]}}{dt}=\text{-V}_{31}\text{+V}_{34}$ (S1.39)

$\frac{d[\text{PTEN]}}{dt}=-\text{V}_{32}\text{+V}_{34}-\text{V}_{35}+\text{V}_{36}-\text{V}_{57}$ (S1.40)

$\frac{d[\text{PIP3]}}{dt}=\text{-V}_{32}\text{-V}_{39}\text{+V}_{56}$ (S1.41)

$\frac{d[\text{PTEN-PIP3]}}{dt}=\text{V}_{32}\text{-V}_{33}$ (S1.42)

$\frac{d[\text{PTEN-PI]}}{dt}=\text{V}_{33}\text{-V}_{34}$ (S1.43)

$\frac{d[\text{pPTEN]}}{dt}=\text{V}_{35}\text{-V}_{36}$ (S1.44)

$\frac{d[\text{PI3K*-PI]}}{dt}=\text{V}_{31}\text{-V}_{55}$ (S1.45)

$\frac{d[\text{PI3K*-PIP3]}}{dt}=\text{V}_{55}\text{-V}_{56}$ (S1.46)

$\frac{d[\text{Akt]}}{dt}=\text{-V}_{39}$ (S1.47)

$\frac{d[\text{pAkt-PIP3]}}{dt}=\text{V}_{40}\text{-V}_{41}\text{-V}_{44}\text{+V}_{47}$ (S1.48)

$\frac{d[\text{Akt-PIP3-PP2A]}}{dt}=\text{V}_{42}\text{-V}_{43}$ (S1.49)

$\frac{d[2C4]}{dt}=\text{-V}_{49}$ (S1.50)

$\frac{d[\text{E2-2C4]}}{dt}=\text{V}_{49}\text{-V}_{50}$ (S1.51)

$\frac{d[\text{E2-2C4}_{\text{c}}\text{]}}{dt}=\text{V}_{50}$ (S1.52)

$\frac{d[\text{PTEN-bpV]}}{dt}=\text{V}_{57}$ (S1.53)

$\frac{d[\text{PI3K-LY]}}{dt}=\text{V}_{58}$ (S1.54)

where reaction rates V_i_ are determined by the following equations:

$\text{V}_{1}\text{=k}_{1}\cdot\text{([E3]}\cdot\text{[HRG]}-\text{K}_{\text{d,1}}\cdot\text{[E3H])}$ (S1.55)

$\text{V}_{2}\text{=k}_{2}\cdot\text{([E3H]}\cdot[\text{E2]}-\text{K}_{\text{d,2}}\cdot[\text{E23H])}$ (S1.56)

$\text{V}_{3}\text{=k}_{3}\cdot\text{([E23H] }-\text{ K}_{e\text{,3}}\cdot[\text{E23HP])}$ (S1.57)

$\text{V}_{4}\text{=V}_{\text{max,4}}\cdot[\text{pE23H] / (K}_{\text{m,4}}\text{ + [pE23H])}$ (S1.58)

$\text{V}_{5}\text{=k}_{5}\cdot\text{([pE23H]}\cdot[\text{Shc] }-\text{ K}_{\text{d,5}}\cdot[\text{pE23H-Shc])}$ (S1.59)

$\text{V}_{6}\text{=k}_{6}\cdot\text{[pE23H-Shc] }-\text{ k}_{\text{-6}}\cdot[\text{pE23H-pShc]}$ (S1.60)

$\text{V}_{7}\text{=k}_{7}\cdot\text{([pE23H-pShc]}\cdot[\text{GS] }-\text{ K}_{\text{d,7}}\cdot[\text{pE23H-pShc-GS])}$ (S1.61)

$\text{V}_{8}\text{=k}_{8}\cdot\text{([pE23H-Shc-GS] }-\text{ K}_{\text{d,8}}\cdot[\text{pE23HP]}\cdot[\text{Shc-GS])}$ (S1.62)

$\text{V}_{9}\text{=k}_{9}\cdot[\text{Shc-GS]}-\text{k}_{\text{-9}}\cdot[\text{pShc]}\cdot[\text{GS]}$ (S1.63)

$\text{V}_{10}\text{=V}_{\text{max,10}}\cdot[\text{pShc] / (K}_{\text{m,10}}\text{ + [pShc])}$ (S1.64)

$\text{V}_{11}\text{=k}_{11}\cdot[\text{Ras-GDP]}\cdot[\text{Shc-GS]/(K}_{\text{m,11}}\text{+[Ras-GDP])}$ (S1.65)

$\text{V}_{12}\text{=V}_{12}\cdot[\text{Ras-GTP]/(K}_{\text{m,12}}\text{+[Ras-GTP])}$ (S1.66)

$\text{V}_{13}\text{=k}_{13}\cdot[\text{Raf]}\cdot[\text{Ras-GTP]/(K}_{\text{m,13}}\text{+[Raf])}$ (S1.67)

$\begin{matrix} \text{V}_{14}\text{=}[\text{Raf*]}\cdot(\text{k}_{14}\cdot\text{[ppAkt-PIP3]/(K}_{m,14}+\text{[Raf*])+} \\ +\text{k}_{\text{14\_1}}\cdot\text{[E}_{\text{Raf}}\text{])/(K}_{\text{m,14\_1}}+\text{[Raf*]))} \end{matrix}$ (S1.68)

$\text{V}_{15}\text{=k}_{15}\cdot[\text{MEK]}\cdot[\text{Raf*]/(K}_{\text{m,15}}\text{+[MEK])}$ (S1.69)

$\text{V}_{16}\text{=k}_{16}\cdot[\text{pMEK]}\cdot[\text{PP2A]}$ (S1.70)

$\text{V}_{17}\text{=k}_{\text{cat,16}}\cdot[\text{pMEK-PP2A]}$ (S1.71)

$\text{V}_{18}\text{=k}_{18}\cdot[\text{MEK-PP2A]}$ (S1.72)

$\text{V}_{19}\text{=k}_{15}\cdot[\text{pMEK]}\cdot[\text{Raf*]/(K}_{\text{m,15}}\text{+[pMEK])}$ (S1.73)

$\text{V}_{20}\text{=k}_{16}\cdot\text{([PP2A]}\cdot[\text{ppMEK] }-\text{K}_{\text{d,16}}\cdot[\text{ppMEK-PP2A])}$ (S1.74)

$\text{V}_{21}\text{=k}_{\text{cat,16}}\cdot[\text{ppMEK-PP2A]}$ (S1.75)

$\text{V}_{22}\text{=k}_{22}\cdot[\text{pMEK-PP2A]}$ (S1.76)

$\text{V}_{23}\text{=k}_{23}\cdot[\text{ERK]}\cdot[\text{ppMEK]/(K}_{\text{m,23}}\text{+[ERK])}$ (S1.77)

$\text{V}_{24}\text{=V}_{\text{max,24}}\cdot[\text{pERK]/(K}_{\text{m,24}}\text{+[pERK])}$ (S1.78)

$\text{V}_{25}\text{=k}_{23}\cdot[\text{ppMEK]}\cdot[\text{pERK]/(K}_{\text{m,23}}\text{+[pERK])}$ (S1.79)

$\text{V}_{26}\text{=V}_{\text{max,24}}\cdot[\text{ppERK]/(K}_{\text{m,24}}\text{+[ppERK])}$ (S1.80)

$\text{V}_{27}\text{=k}_{27}\cdot\text{([pE23H]}\cdot[\text{PI3K] }-\text{K}_{\text{d,27}}\cdot[\text{pE23H-PI3K])}$ (S1.81)

$\text{V}_{28}\text{=k}_{28}\cdot[\text{pE23H-PI3K]}-k_{-28}\cdot[\text{pE23H-PI3K*] }$ (S1.82)

$\text{V}_{29}\text{=k}_{29}\cdot[\text{pE23H-PI3K*]}-\text{k}_{\text{-29}}\cdot[\text{pE23H}\cdot\text{PI3K*]}$ (S1.83)

$\text{V}_{30}\text{=k}_{30}\cdot[\text{PI3K*]}$ (S1.84)

$\text{V}_{31}\text{=k}_{31}\cdot\text{([PI]}\cdot[\text{PI3K*]}-\text{K}_{\text{d,31}}\cdot[\text{PI3K*-PI])}$ (S1.85)

$\text{V}_{32}\text{=k}_{32}\cdot\text{([PIP3]}\cdot[\text{PTEN]}-\text{K}_{\text{d,32}}\cdot[\text{PTEN-PIP3])}$ (S1.86)

$\text{V}_{33}\text{=k}_{33}\cdot[\text{PTEN-PIP3]}$ (S1.87)

$\text{V}_{34}\text{=k}_{34}\cdot\text{[PTEN-PI] }$ (S1.88)

$\text{V}_{35}\text{=V}_{\text{max,35}}\cdot[\text{PTEN]/(K}_{\text{m,35}}\text{+[PTEN])}$ (S1.89)

$\text{V}_{36}\text{=V}_{\text{m\_35}}\cdot[p\text{PTEN]/(K}_{\text{m\_35}}\text{+[}p\text{PTEN])}$ (S1.90)

$\text{V}_{39}\text{=k}_{39}\cdot\text{([PIP3]}\cdot[\text{Akt]}-\text{K}_{\text{d,39}}\cdot[\text{Akt-PIP3])}$ (S1.91)

(S1.92)

(S1.93)

$\text{V}_{42}\text{=k}_{\text{cat,42}}\cdot[\text{pAkt-PIP3-PP2A]}$ (S1.94)

$\text{V}_{43}\text{=k}_{43}\cdot[\text{Akt-PIP3-PP2A]}$ (S1.95)

(S1.96)

(S1.97)

(S1.98)

$\text{V}_{47}\text{=k}_{47}\cdot[\text{pAkt-PIP3-PP2A]}$ (S1.99)

$\text{V}_{48}\text{=k}_{48}\cdot[\text{pE23H]}$ (S1.100)

$\text{V}_{49}\text{=k}_{49}\cdot\text{([2C4]}\cdot[\text{E2]}-\text{K}_{\text{d,49}}\cdot[\text{E2-2C4])}$ (S1.101)

$\text{V}_{50}\text{=k}_{50}\cdot[\text{E2-2C4]}-\text{k}_{\text{-50}}\cdot[\text{E2-2C4}_{\text{c}}\text{]}$ (S1.102)

$\text{V}_{51}\text{=k}_{51}\cdot[\text{E3H]}$ (S1.103)

$\text{V}_{52}\text{=k}_{2}\cdot\text{([E3H}_{\text{c}}]\cdot[\text{E2]}-\text{K}_{\text{d,2}}\cdot[\text{E23H])}$ (S1.104)

$\text{V}_{53}\text{=k}_{53}\cdot[\text{E23H]}$ (S1.105)

$\text{V}_{54}\text{=k}_{3}\cdot\text{([E23H}_{\text{c}}]-\text{ K}_{\text{d,3}}\cdot[\text{pE23H])}$ (S1.106)

$\text{V}_{55}\text{=k}_{55}\cdot[\text{PI3K*-PI]}$ (S1.107)

$\text{V}_{56}\text{=k}_{56}\cdot[\text{PI3K*-PIP3]}$ (S1.108)

$\text{V}_{57}\text{=k}_{57}\cdot\text{([PTEN}]\cdot[\text{bpV}]-\text{ K}_{\text{d,57}}\cdot[\text{PTEN\_bpV])}$ (S1.109) $\text{V}_{58}\text{=k}_{58}\cdot\text{([PI3K]}\cdot[\text{LY}]-\text{ K}_{\text{d,58}}\cdot[\text{PI3K\_LY])}$ (S1.110)

###

### Supplementary Table S1. List of species used in the ErbB2/3 network model and their abbreviations in the text, diagrams and SBML file

| **Abbreviations used in the text, ODEs and schemes** | **Protein names** | **Abbreviations in SBML file** |
| --- | --- | --- |
| E2, HER2, ErbB2 | ErbB2 receptor | E2 |
| E3, HER3, ErbB3 | ErbB3 receptor | E3 |
| HRG | Heregulin | HRG |
| E3H | ErbB3/HRG intermediate ligand-receptor complex | E3H |
| E3H_c_ | ErbB3/HRG final ligand-receptor complex | E3H_C |
| E23H | Heterodimer of ErbB2 and ErbB3/HRG, intermediate form | E23H |
| E23H_c_ | Heterodimer of ErbB2 and ErbB3/HRG, final form | E23H_C |
| pE23H | Phosphorylated form of heterodimer of ErbB2/ErbB3 bound with HRG | E23HP |
| Grb2 | growth factor receptor-binding protein 2 |  |
| Ras-GDP | Ras-GDP protein | RasGDP |
| Ras-GTP | Ras-GTP protein | RasGTP |
| Raf | Raf protein | Raf |
| Raf* | Activated Raf | Rafa |
| PTEN | Phosphatase and tensinhomologue deleted on chromosome ten | PTEN |
| pPTEN, PTENP | Phosphorylated PTEN | PTENP |
| Akt | RAC serine/threonine-protein kinase | Akt |
| Per, 2C4 | Pertuzumab | Per |
| MAPK | Mitogen-activated protein kinase |  |
| MEK | MAPK/ERK kinase |  |
| pMEK | Phosphorylated MEK | MEKP |
| ppMEK | Doubly phosphorylated MEK | MEKPP |
| ERK | extracellular signal-regulated kinase |  |
| pERK | Phosphorylated ERK | ERKP |
| ppERK | Doubly phosphorylated ERK | ERKPP |
| MKP3 | MAPK phosphatase 3 |  |
| PDK1 | 3-phosphoinositide-dependent kinase 1 |  |
| PI, PI2 | phosphatidylinositol-4,5-bisphosphate | PI |
| PIP3 | phosphatidylinositol-3,4,5-trisphosphate |  |
| PI3K | phosphatidylinositol 3’-kinase | PI3K |
| PI3K* | Activated PI3K | PI3Ka |
| PP2A | protein phosphatase 2A | PP2A |
| Shc | src homology 2 domain-containing transforming protein C | Shc |
| pShc | Phosphorylated Shc | ShcP |
| Sos | Son of Sevenless protein |  |
| GS | Grb2–Sos complex | GS |
| pE23H-Shc | Complex of pE23H with Shc | E23HP_Shc |
| pE23H-pShc | Complex of pE23H with pShc | E23HP_ShP |
| Shc-GS | Complex of Shc with GS | ShGS |
| pE23H-pShc-GS | Complex of pE23H-pShc with GS | E23HP_ShGS |
| AKT-PIP3 | Complex of AKT with PIP3 | AKT_PIP3 |
| pAKT-PIP3 | Complex of pAKT with PIP3 | AKT_PI_P |
| ppAKT-PIP3 | Complex of ppAKT with PIP3 | AKT_PI_PP |
| ppAKT-PIP3-PP2A | Complex of ppAKT-PIP3 with PP2A | AKT_PI_PP_PP2A |
| AKT-PIP3-PP2A | Complex of AKT-PIP3 with PP2A | AKT_PIP3_PP2A |
| pAKT-PIP3-PP2A | Complex of pAKT-PIP3 with PP2A | AKT_PI_P_PP2A |
| E2-2C4 | Intermediate complex of ErbB2 with pertuzumab | E2_Per |
| E2-2C4_c_ | Complex of ErbB2 with pertuzumab | E2Per |
| PTEN-PIP3 | Complex of PTEN with PIP3 | PTEN_PIP3 |
| PTEN-PI | Complex of PTEN with PI | PTEN_PI |
| pMEKP-PP2A | Complex of pMEKP with PP2A | MEKP_PP2A |
| MEK-PP2A | Complex of MEK with PP2A | MEK_PP2A |
| ppMEKPP-PP2A | Complex of ppMEKPP with PP2A | MEKPP_PP2A |
| pE23H-PI3K | Complex of pE23H with PI3K | E23HP_PI3K |
| PI3K*-PI | Complex of PI3K* with PI | PI3Ka_PI |
| pE23H-PI3K* | Complex of pE23H with PI3K* | E23HP_PI3Ka |
| E_Raf_ | An enzyme dephosphorylating Raf* | E_Raf |
| bpV | Bisperoxovanadium compound, bpV(pic), PTEN inhibitor | bpV |
| LY | LY294002, PI3K inhibitor | LY |
| PTEN-bpV | Enzyme-inhibitor complex of PTEN and bpV(pic) | PTEN_bpV |
| PI3K-LY | Enzyme-inhibitor complex of PI3K and LY294002 | PI3K_LY |

### Identifiability analysis of ErbB2/ErbB3 network model

We have run identifiability analysis of the model in SBTOOLBOX2.0 (www.sbtoolbox2.org). Our model was not fully identifiable as some parameters were highly correlated with each other. The subset of parameters that were correlated at 95% confidence is shown in the Supplementary Figure S6. None of the parameters listed in the diagram, could be uniquely estimated with the use of the time series data we had (Faratian *et al*, 2009). To achieve better identifiability , some of these parameters would have to be measured independently (e.g. affinity constants), and be fixed in the model, so the rest could be identified from fitting the model to time series data.

Comparison of the Figure S6 with the pAkt sensitivity spectrum presented in Figure 2 shows that the set of non-identifiable parameters overlaps with the list of the most sensitive parameters in the model. The only parameter out of 20 shown on Figure S7, which is missing on figure 2, is k47 (a rate constant in PP2A catalytic cycle), that proves again that our global sensitivity analysis captures the sensitive core of the model properly.

One of the satisfactory fittings of the model to experimental data is shown in Supplementary Figure S7. The nominal model parameter values used for model simulation are given in Supplementary table S2.


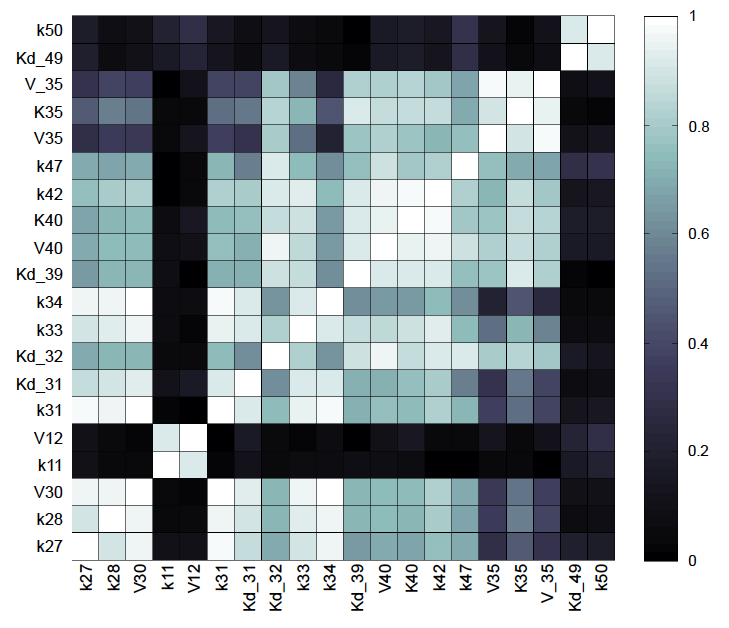


### Supplementary Figure S5. Non-identifiable parameters of ErbB2/3 network model.

Correlation matrix for non-identifiable ErbB2/3 model parameters (95% confidence)

| A | pErbB3 |
| --- | --- |
| B | pAkt |
| C | pErk    Time, min |

### Supplementary Figure S6: ErbB2/3 model calibration

An example of a satisfactory fitting of the ErbB2/3 model to experimental data: simulated (lines) and experimental (points) time-course profiles of phosphorylated proteins in PE04 cancer cell line after stimulation with heregulin-β, in the absence (black) and presence (blue) of pertuzumab. The data were normalised to the maximum of the signal observed in the absence of Pertuzumab. A - total pErbB3; B - total pAkt; C - total pErk.
