# Supplementary material for: Model-based global sensitivity analysis as applied to identification of anti-cancer drug targets and biomarkers of drug resistance in the ErbB2/3 network
Source: Eur J Pharm Sci. 2012 Jul 16;46(4):244–58. doi: 10.1016/j.ejps.2011.10.026 (PMC3398788; doi:10.1016/j.ejps.2011.10.026)
Supplement: Supplementary data 3 [file mmc3.doc]

**Additional file 3**

**Supplementary information on GSA analysis of ErbB2/ErbB3 network model**

**Table of contents**

Supplementary Figure S7. Distribution of parameter values in the set of Sobol points. [2](#__RefHeading___Toc169582882)

Supplementary Figure S8. Association between model output and selected input parameters. [8](#__RefHeading___Toc169582883)

Supplementary Figure S9. Definition of the optimal sample size N for GSA of ErbB2/3 network model. [11](#__RefHeading___Toc169582884)

Supplementary Figure S10. Global sensitivity profile of [12](#__RefHeading___Toc169582885)

Analysis of the changes in the sensitivity spectrum of pAkt signal, caused by the introduction of anti-ErbB2 inhibitor pertuzumab. [13](#__RefHeading___Toc169582886)

Supplementary figure S11. Correlation between PP2A expression and cell growth inhibition by pertuzumab [14](#__RefHeading___Toc169582887)

References [15](#__RefHeading___Toc169582888)


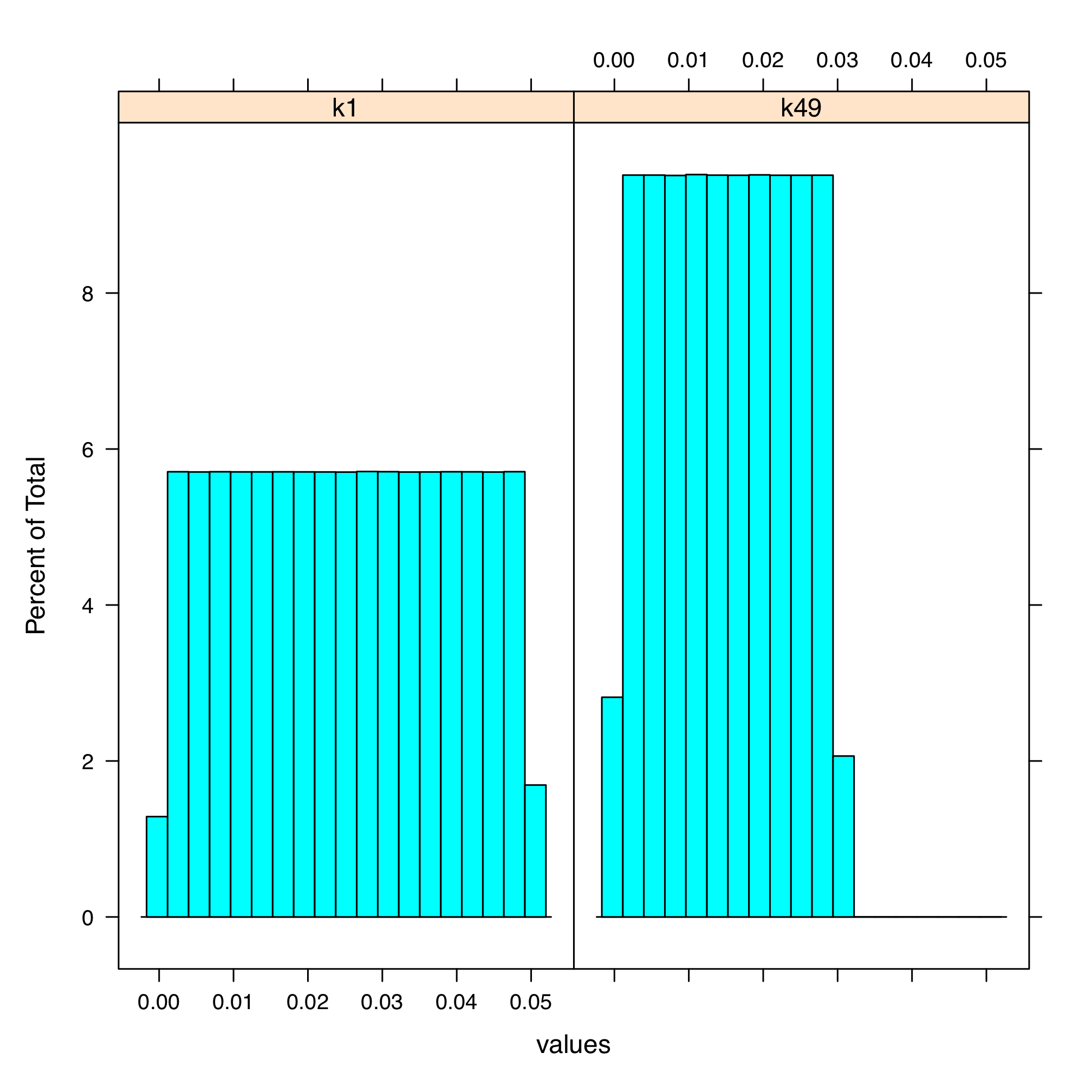

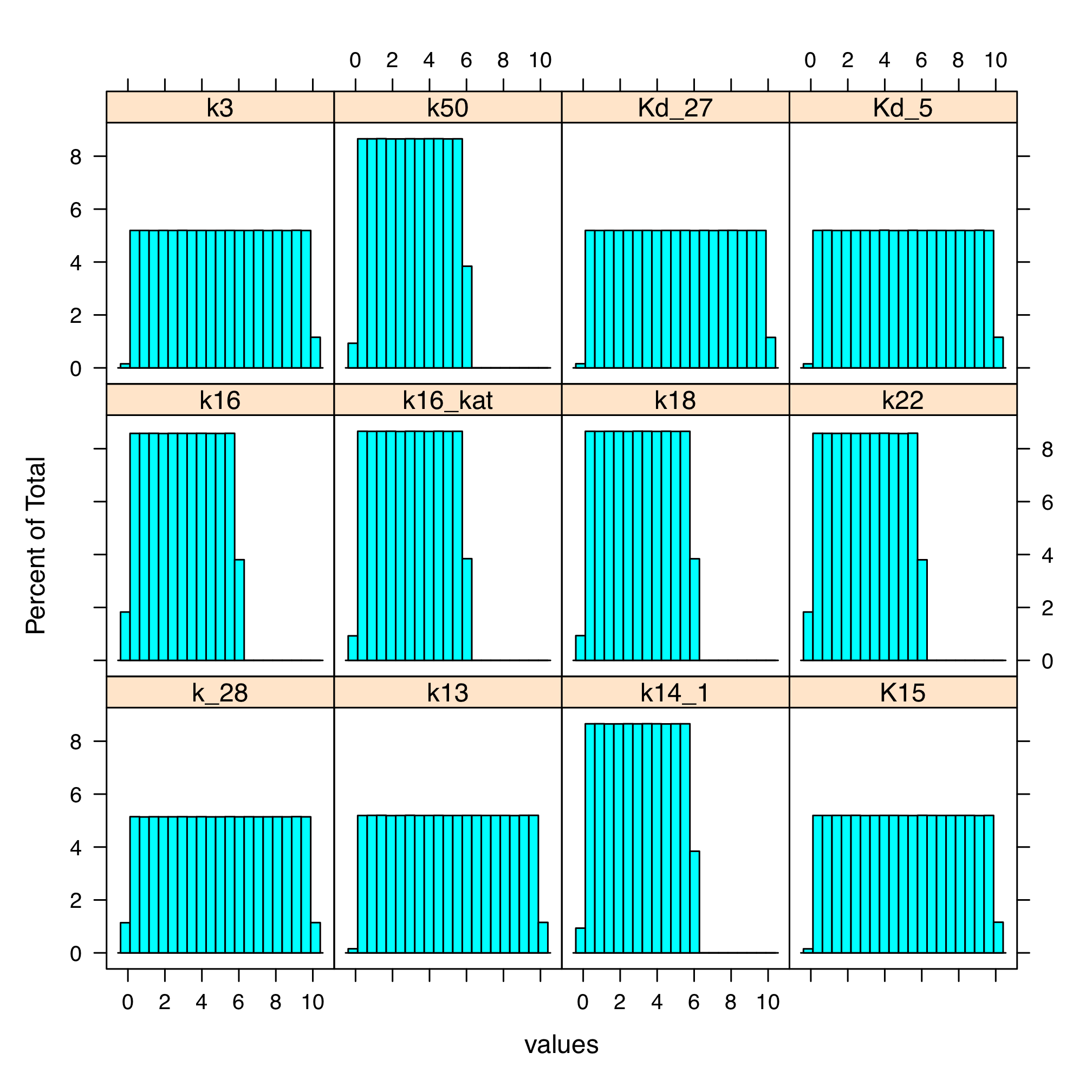


### Supplementary Figure S7. Distribution of parameter values in the set of Sobol points.

Frequency distributions of parameter values in the set of Sobol points, sampled from the hypercube defined by parameter ranges indicated in Additional file 2. The total number of parameter variants in each distribution is 120000.


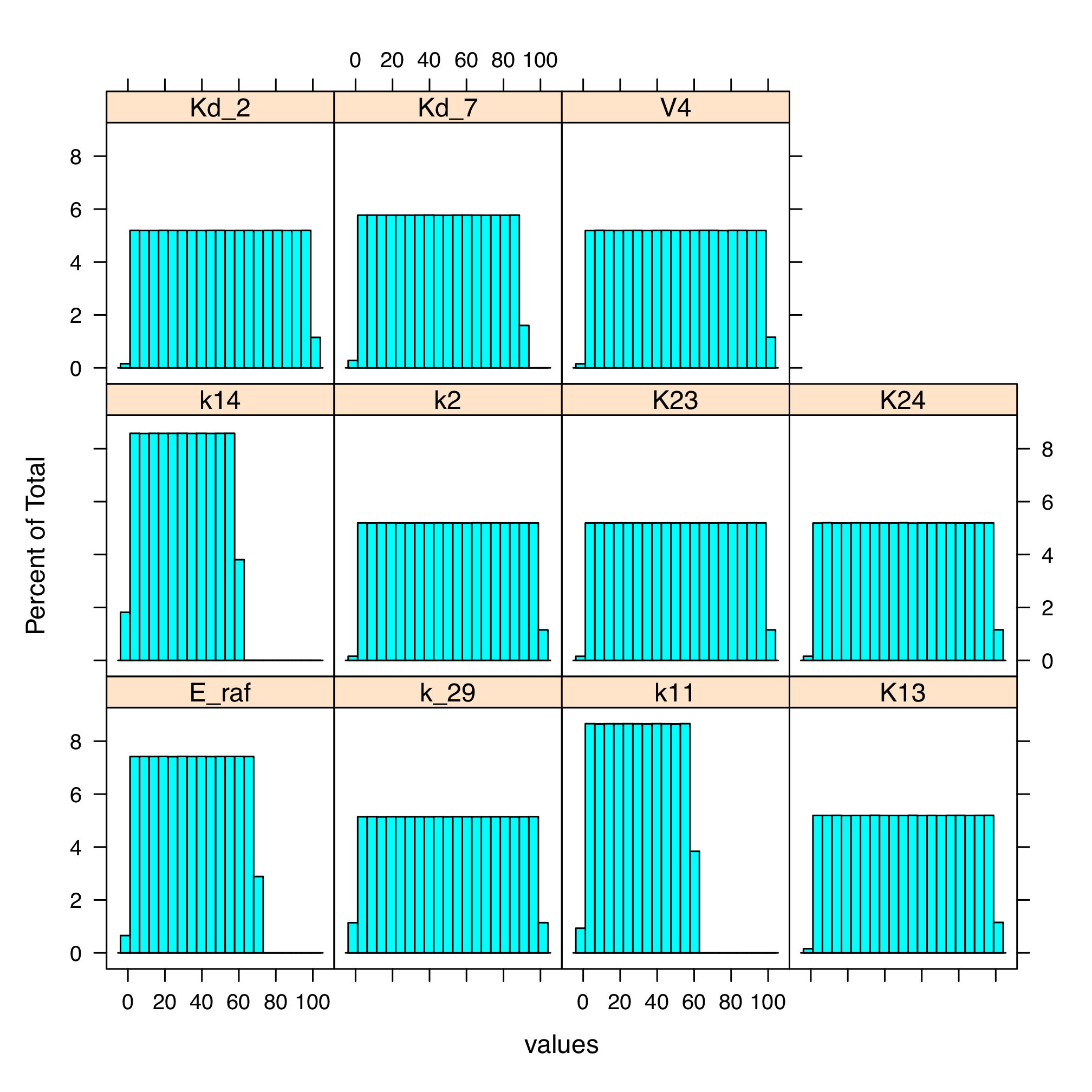

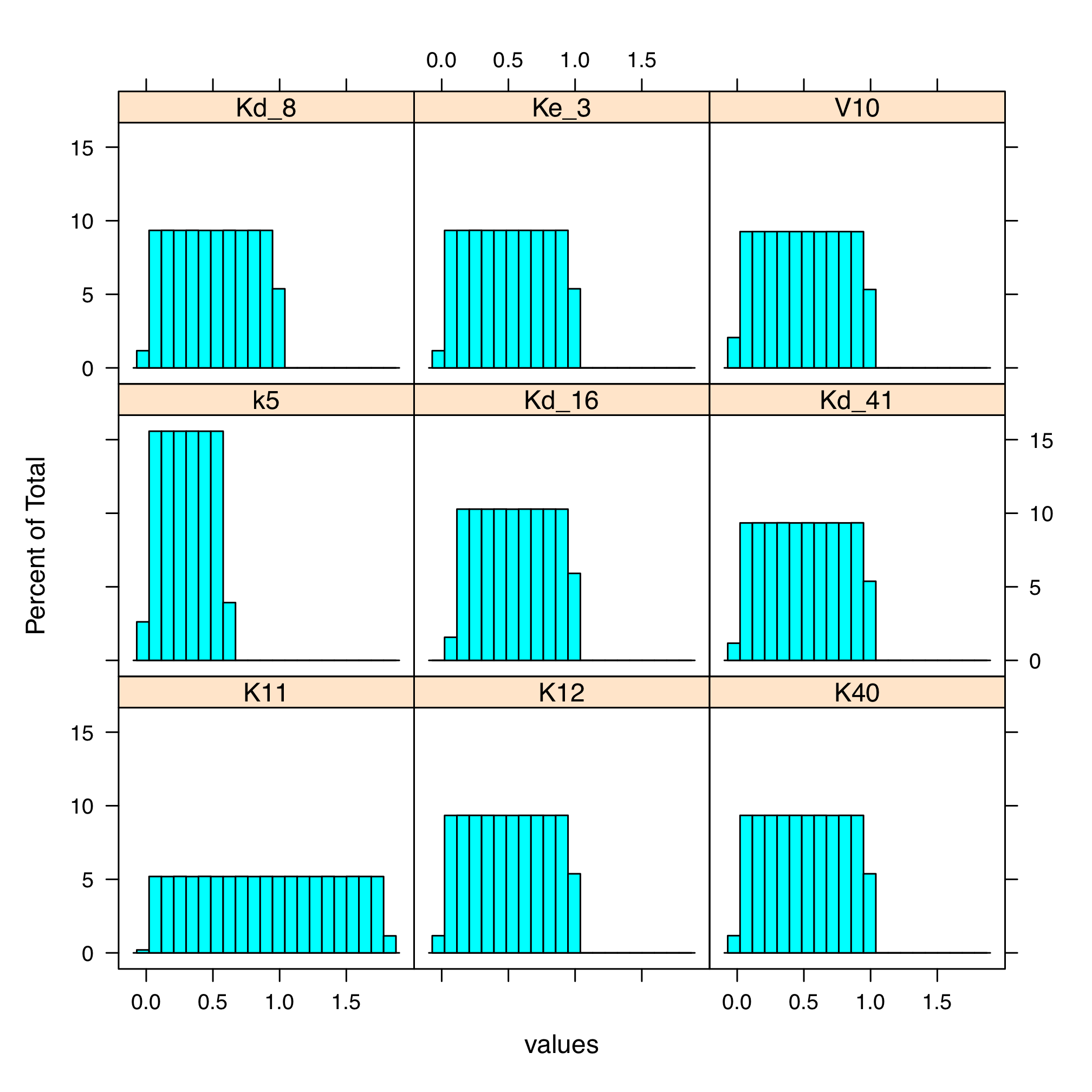


Supplementary Figure S7 (continued)


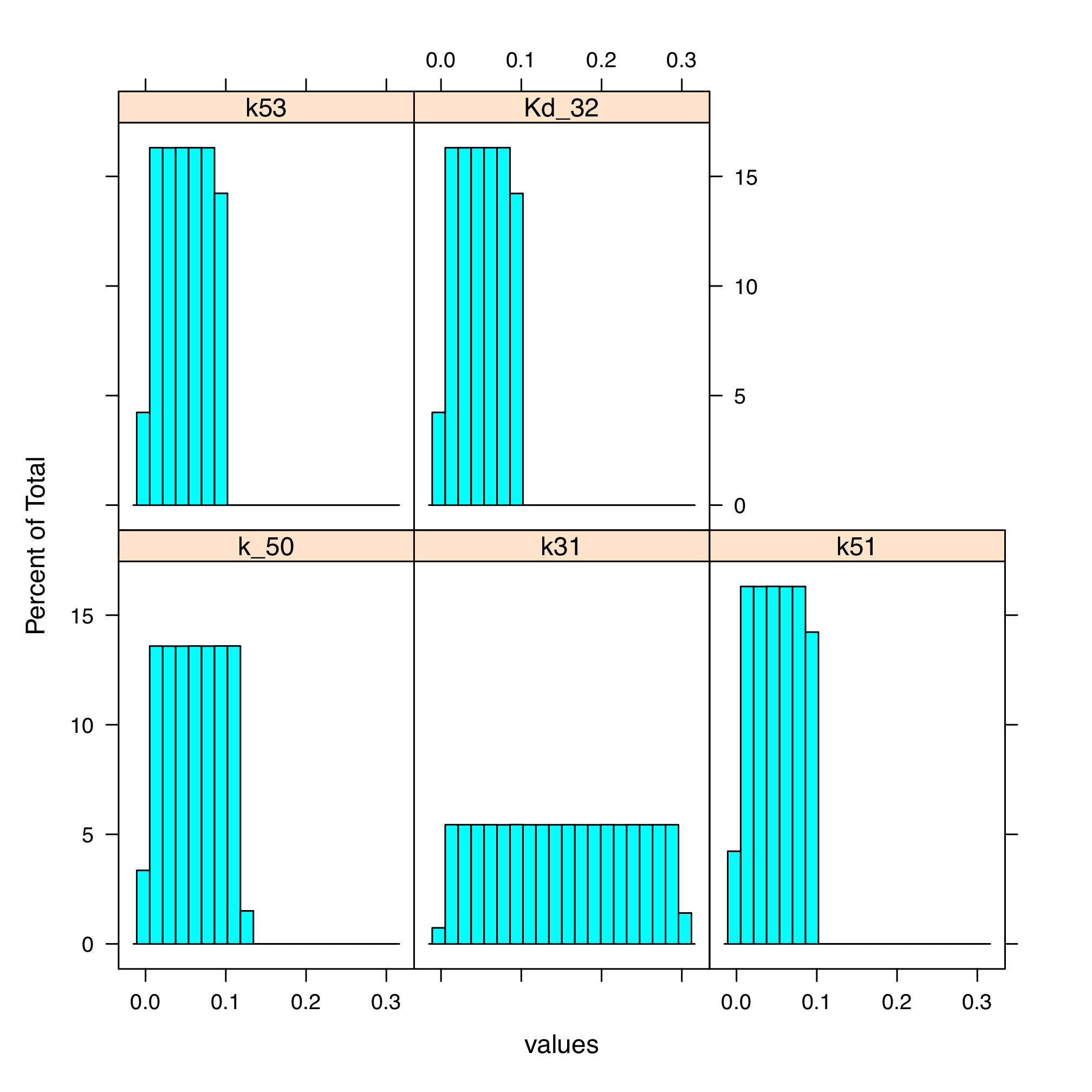

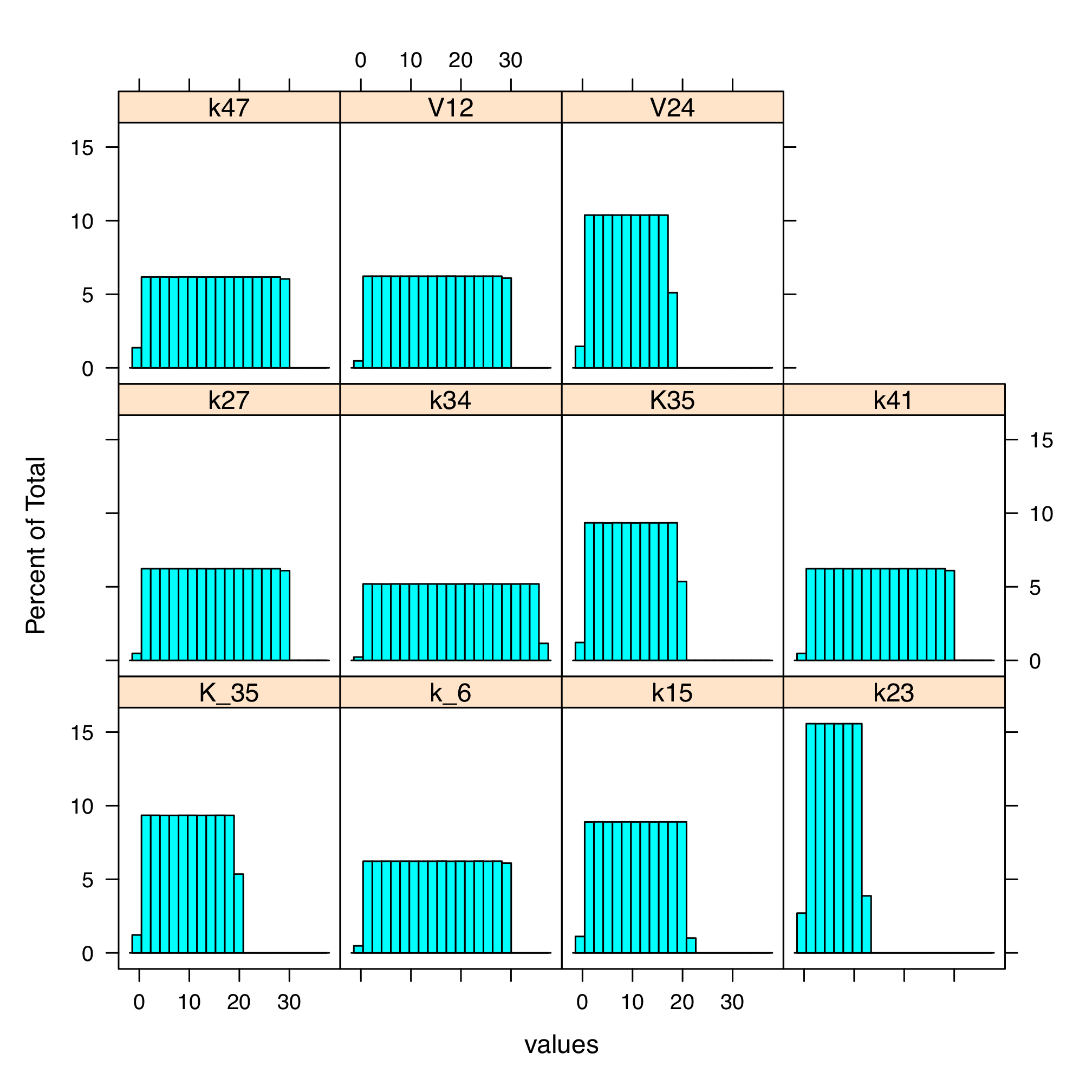
 Supplementary Figure S7 (continued)


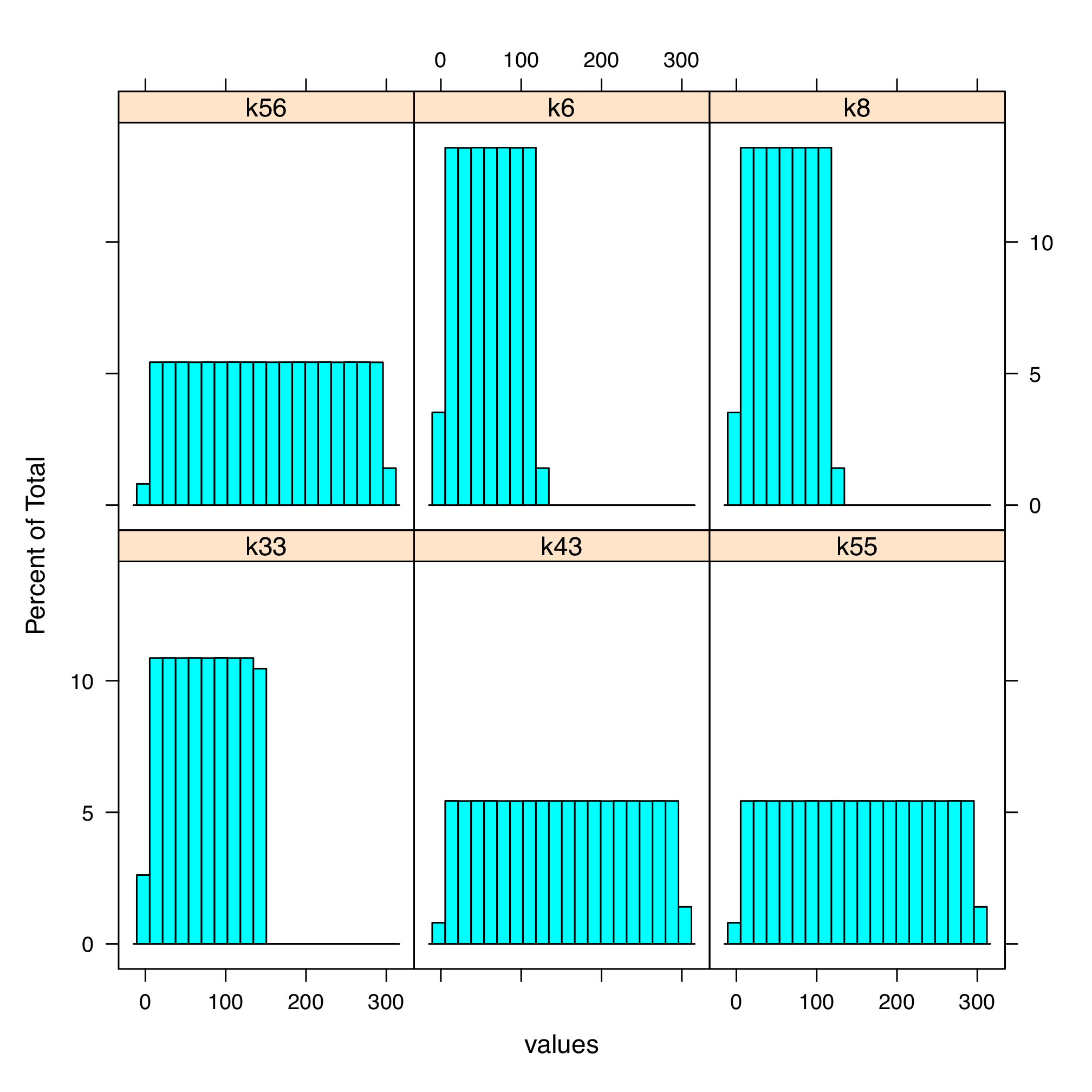

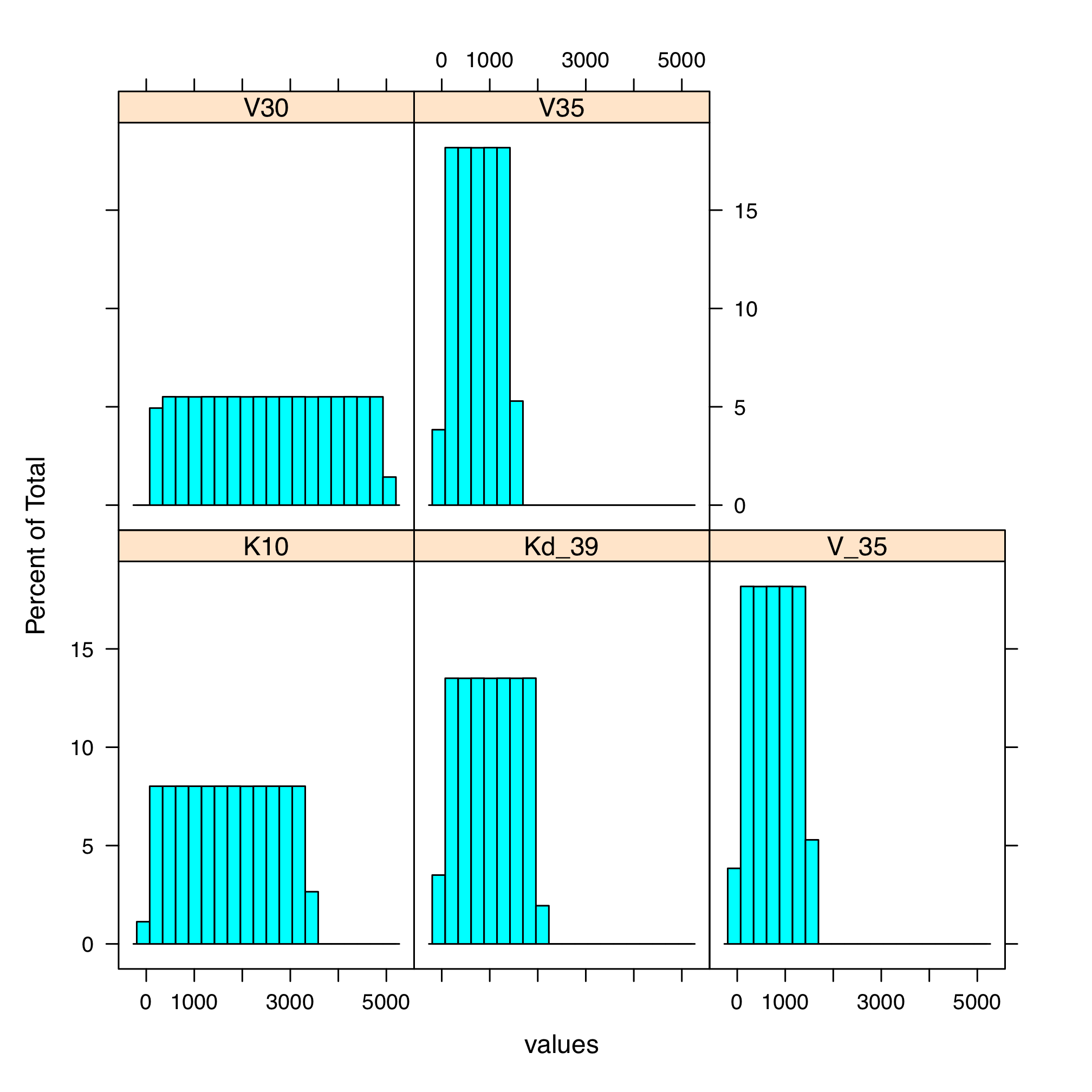


Supplementary Figure S7 (continued)


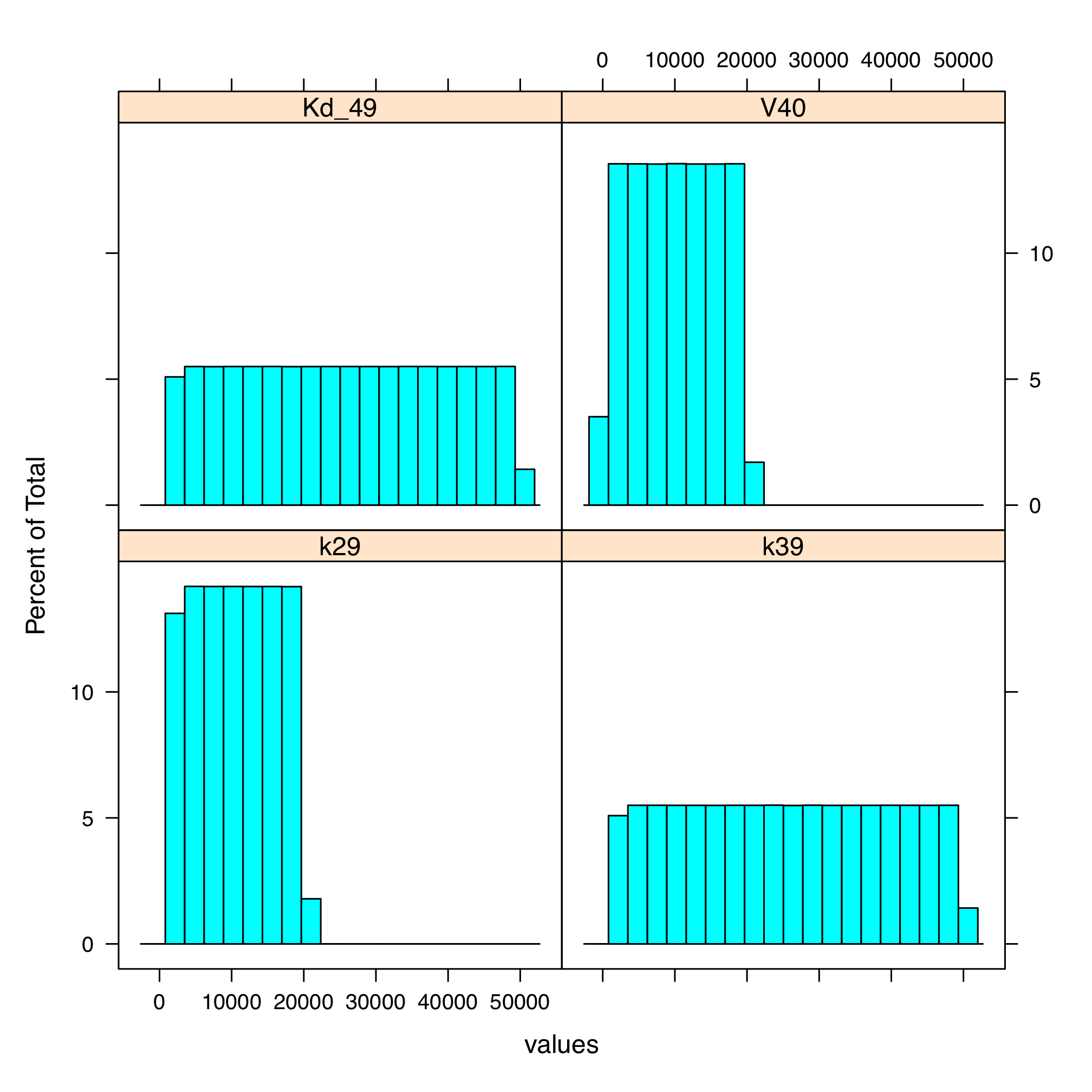

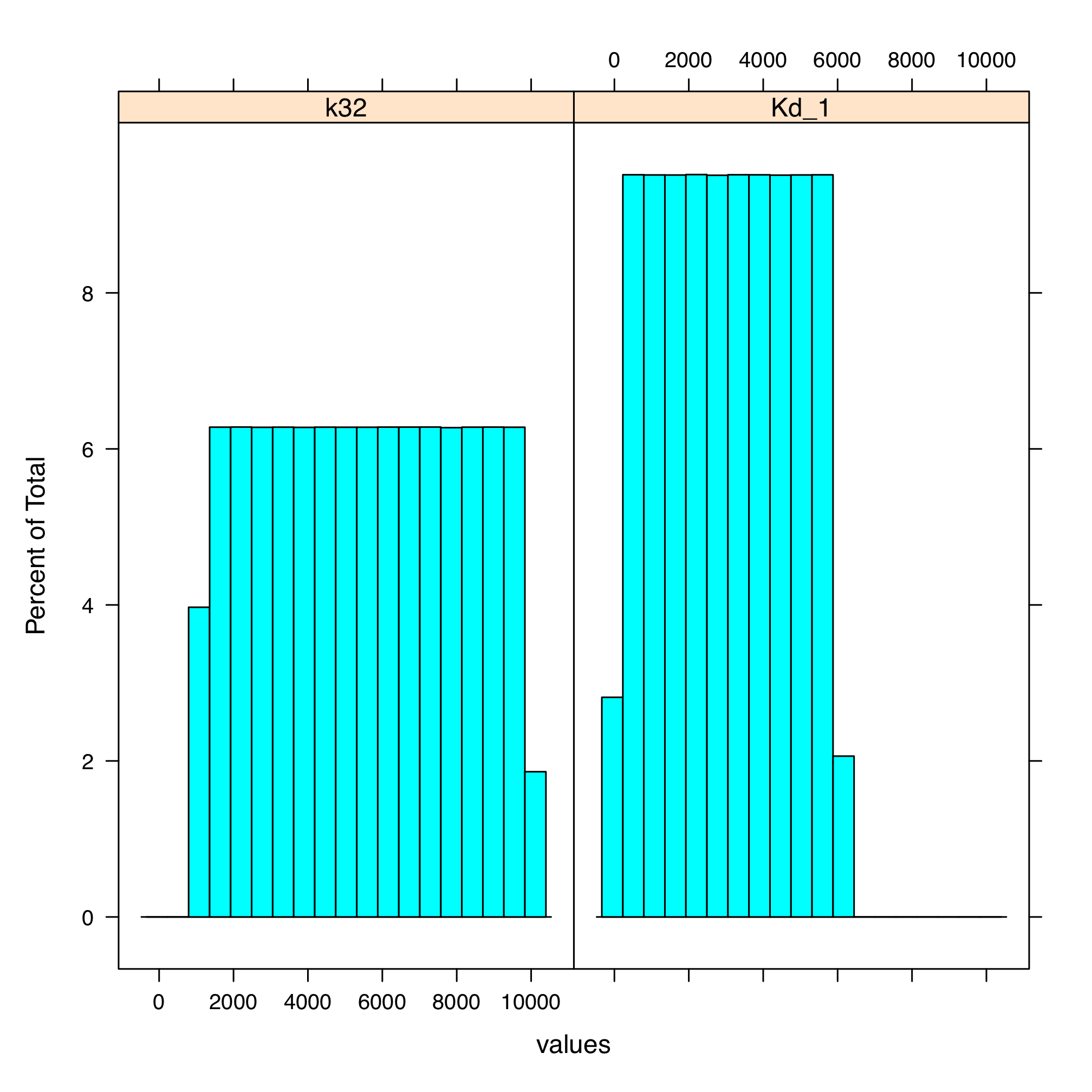

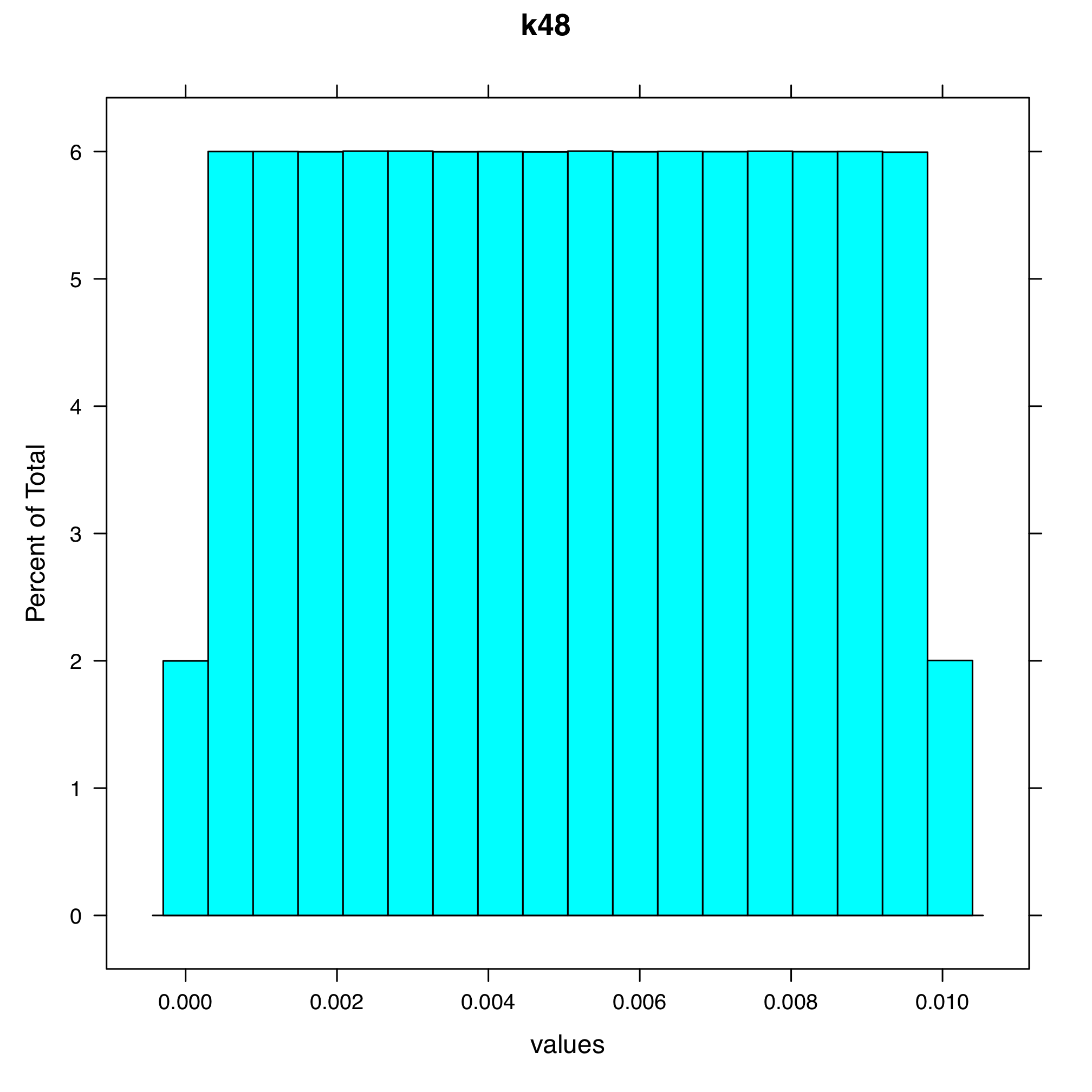


Supplementary Figure S7 (continued)


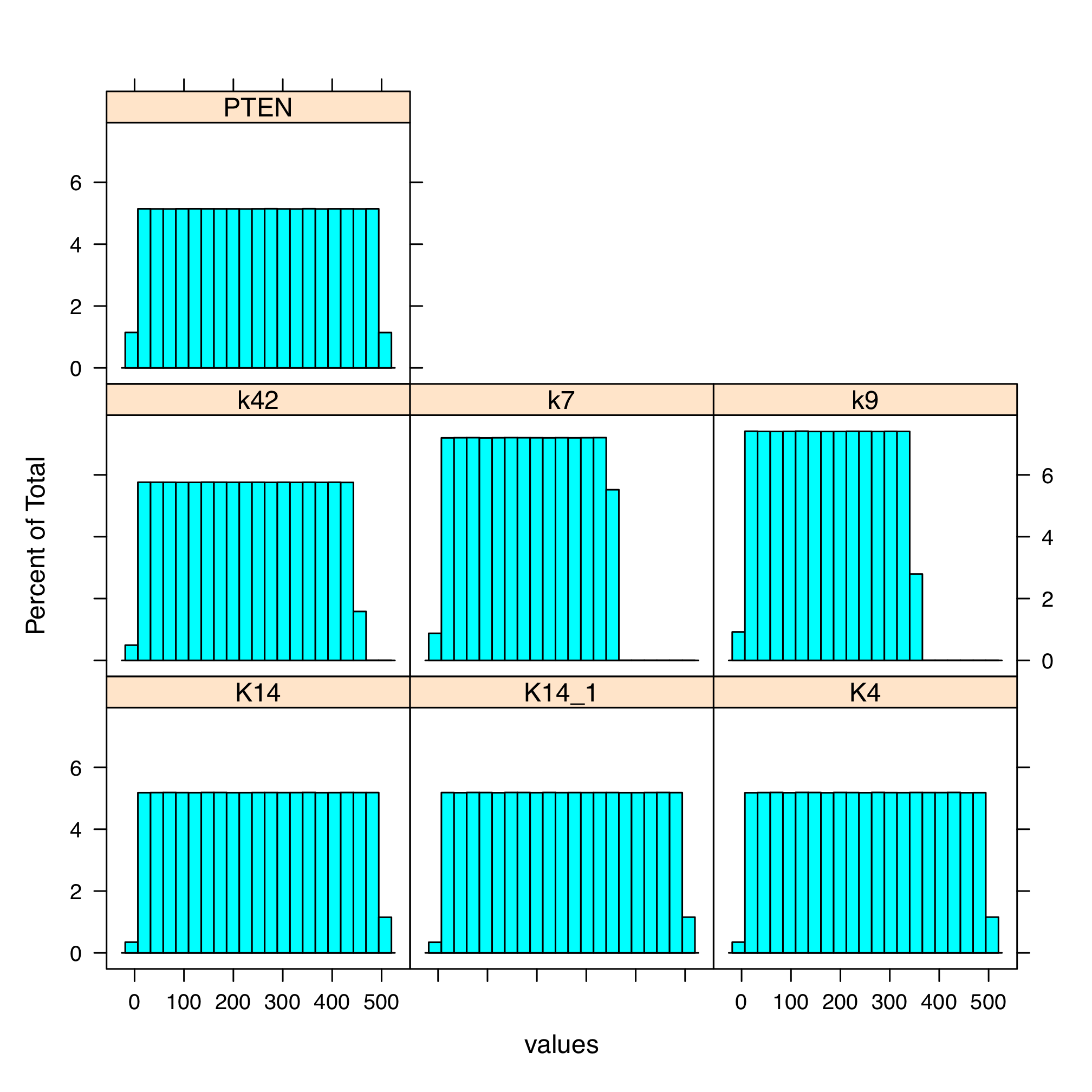

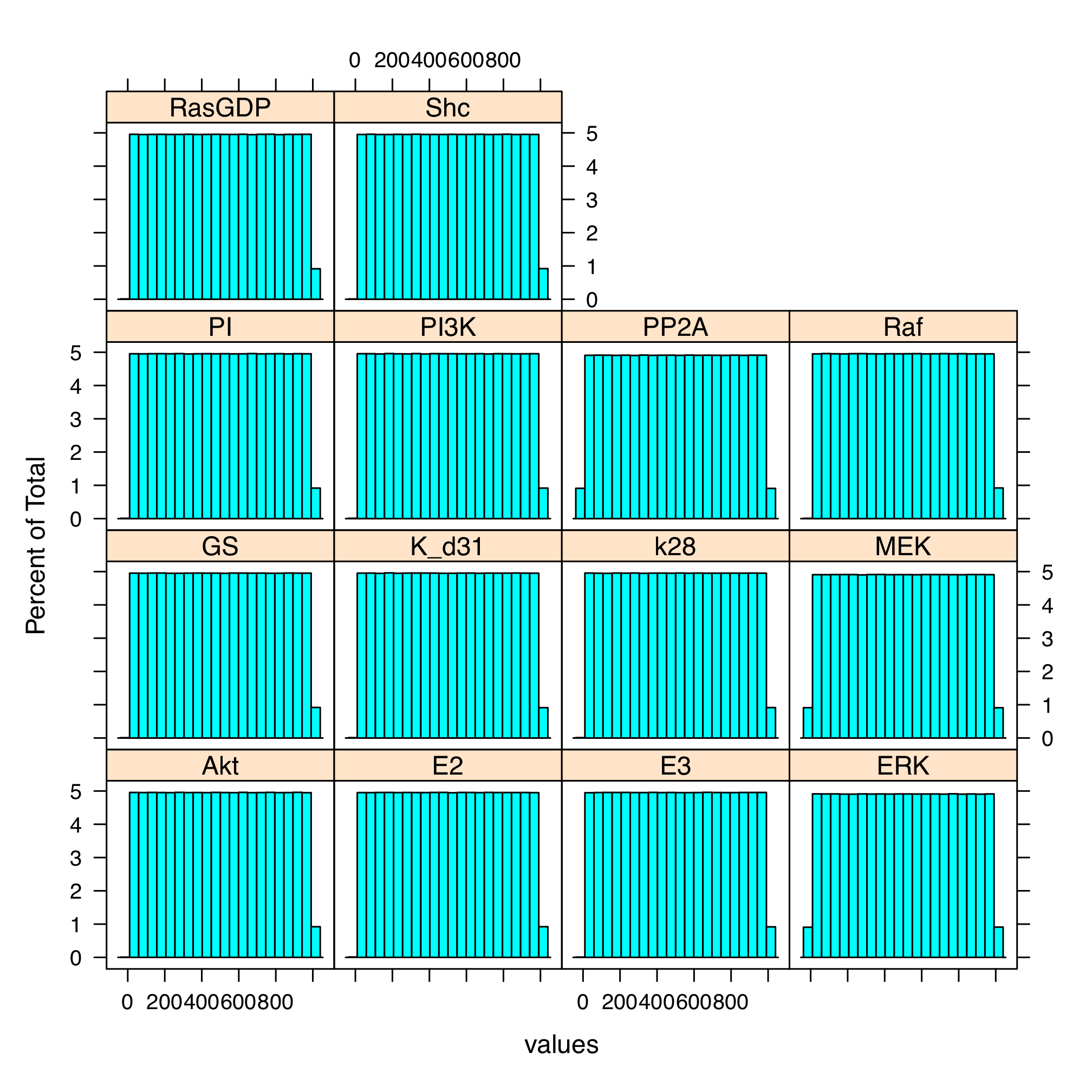


Supplementary Figure S7 (continued)


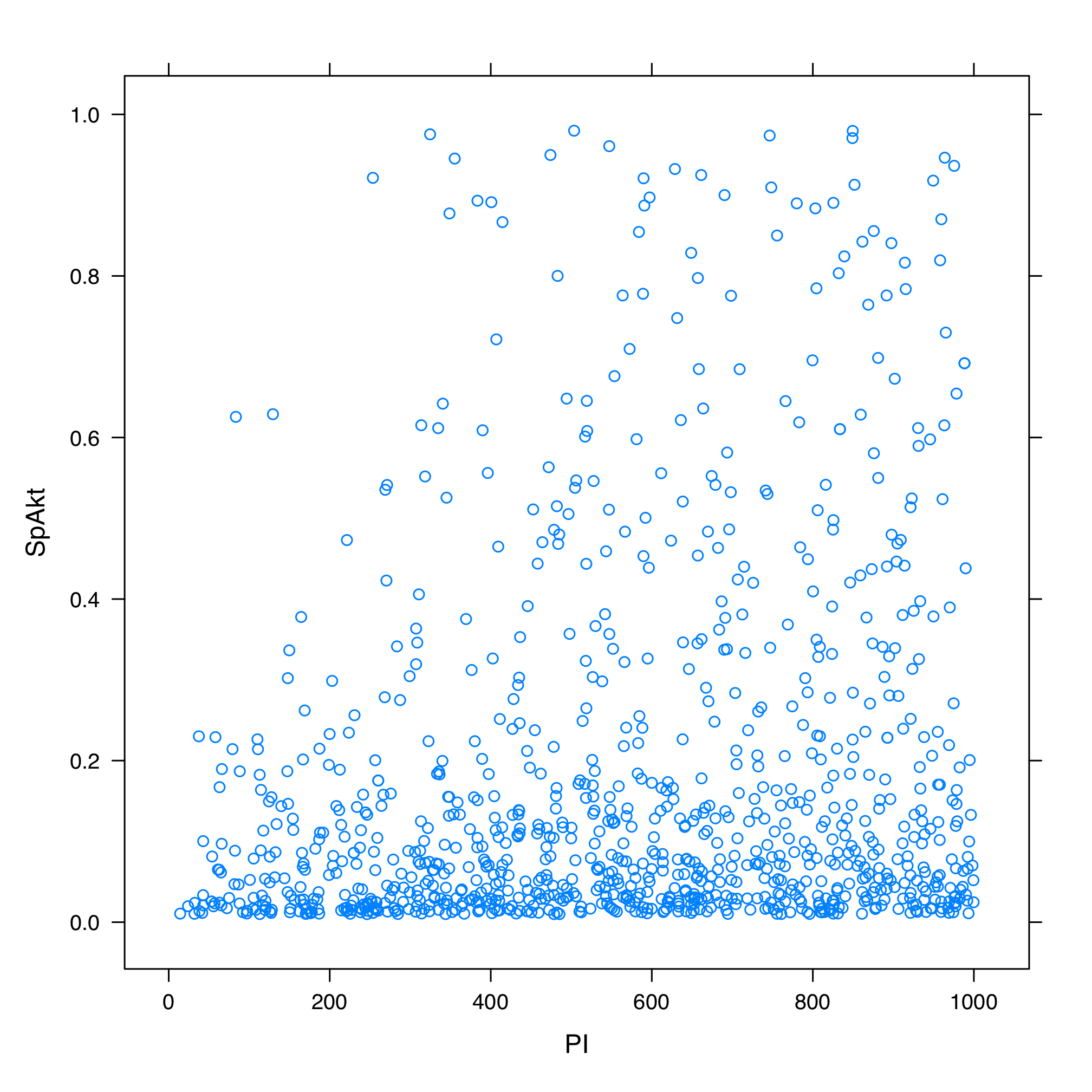

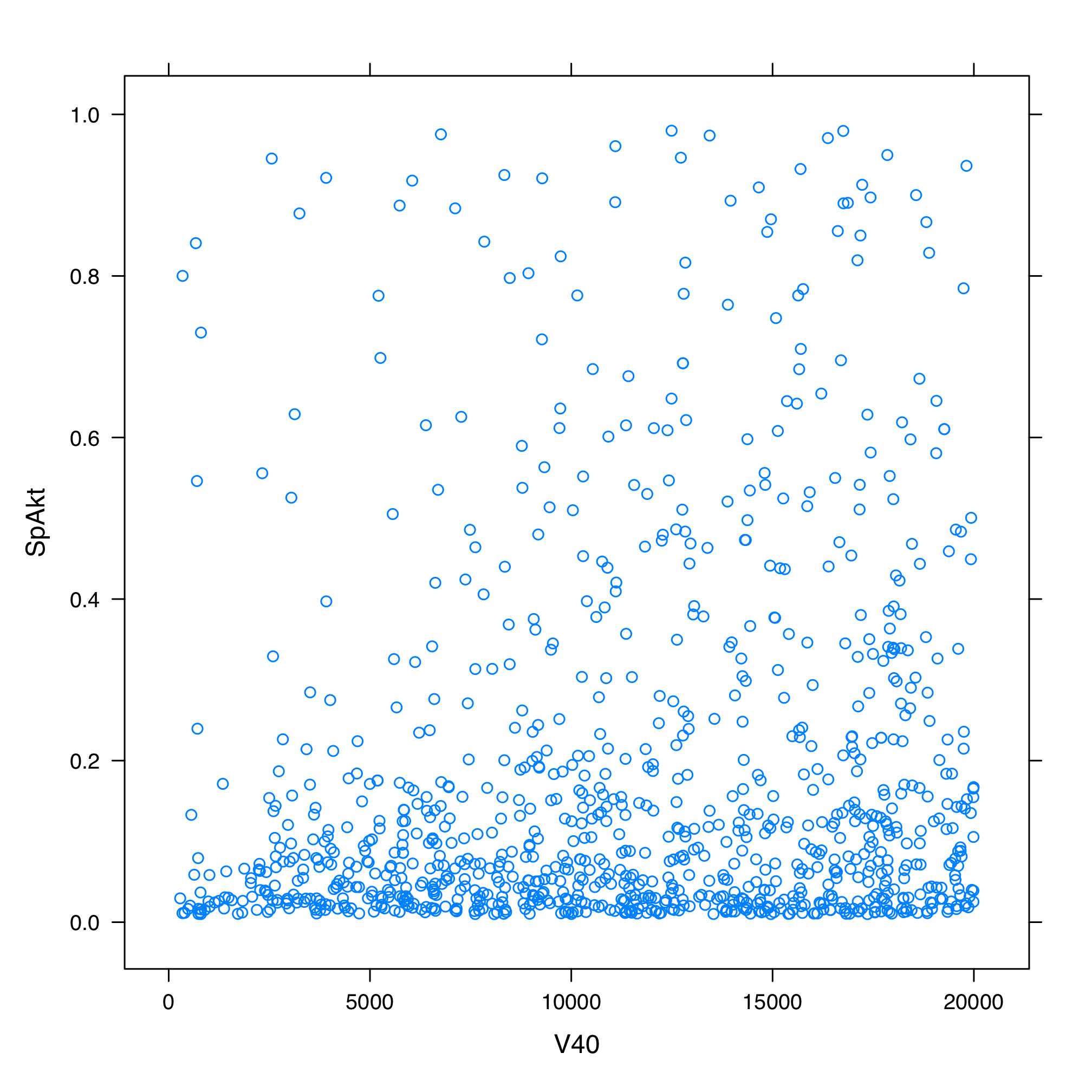


### Supplementary Figure S8. Association between model output and selected input parameters.

The reference output is the area-under-the-time-course-profile of phosphorylated Akt (S_pAkt_ ) and it is shown on the ordinate. Input parameters of interest are represented on the abscissa. Each dot represents the calculated output value S_pAkt_ for a specific sampled value of the parameter. To make the graphs less busy we only present the data for sample size N=1000. However the similar trend was observed for larger sample sizes, including N=120000, used in our final analysis


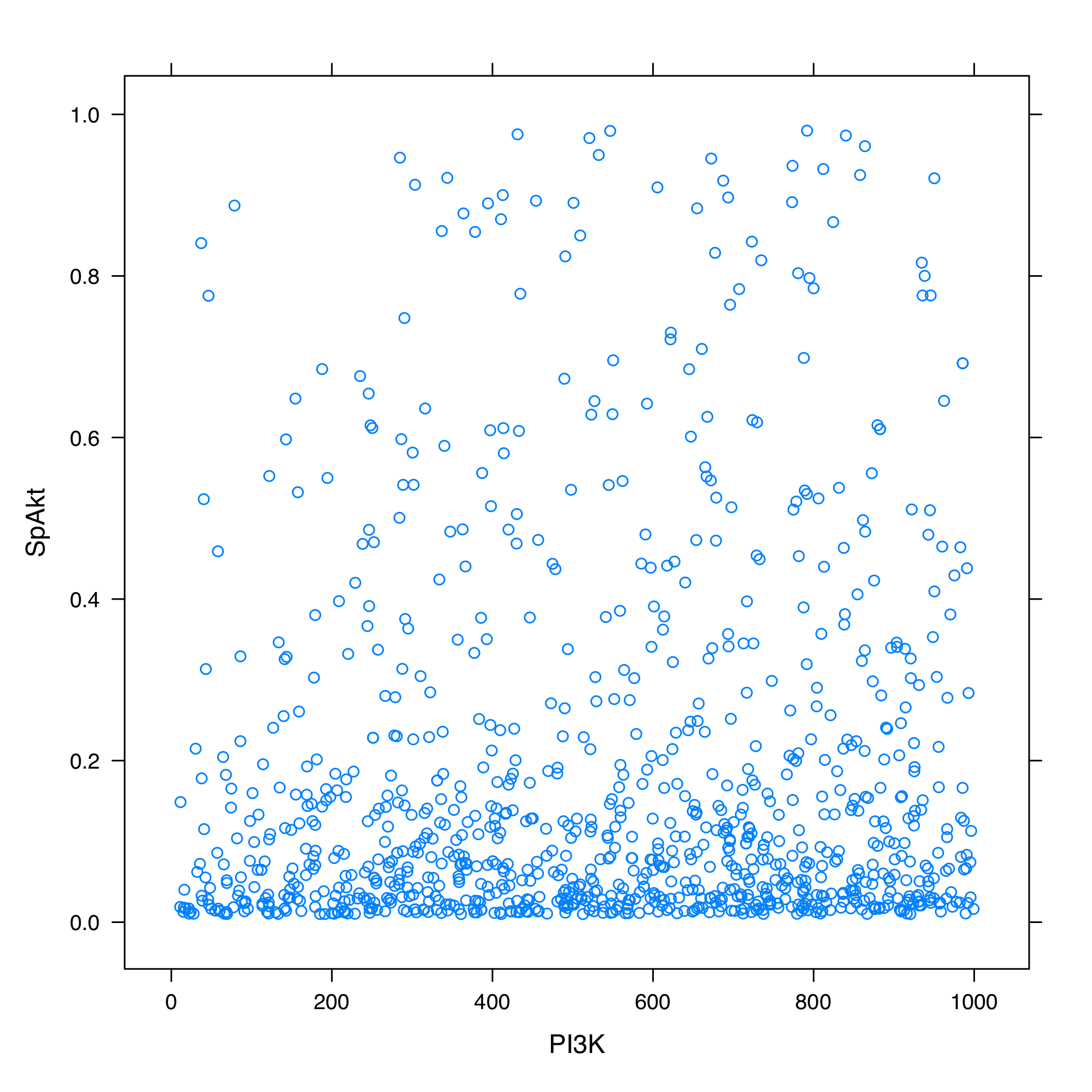

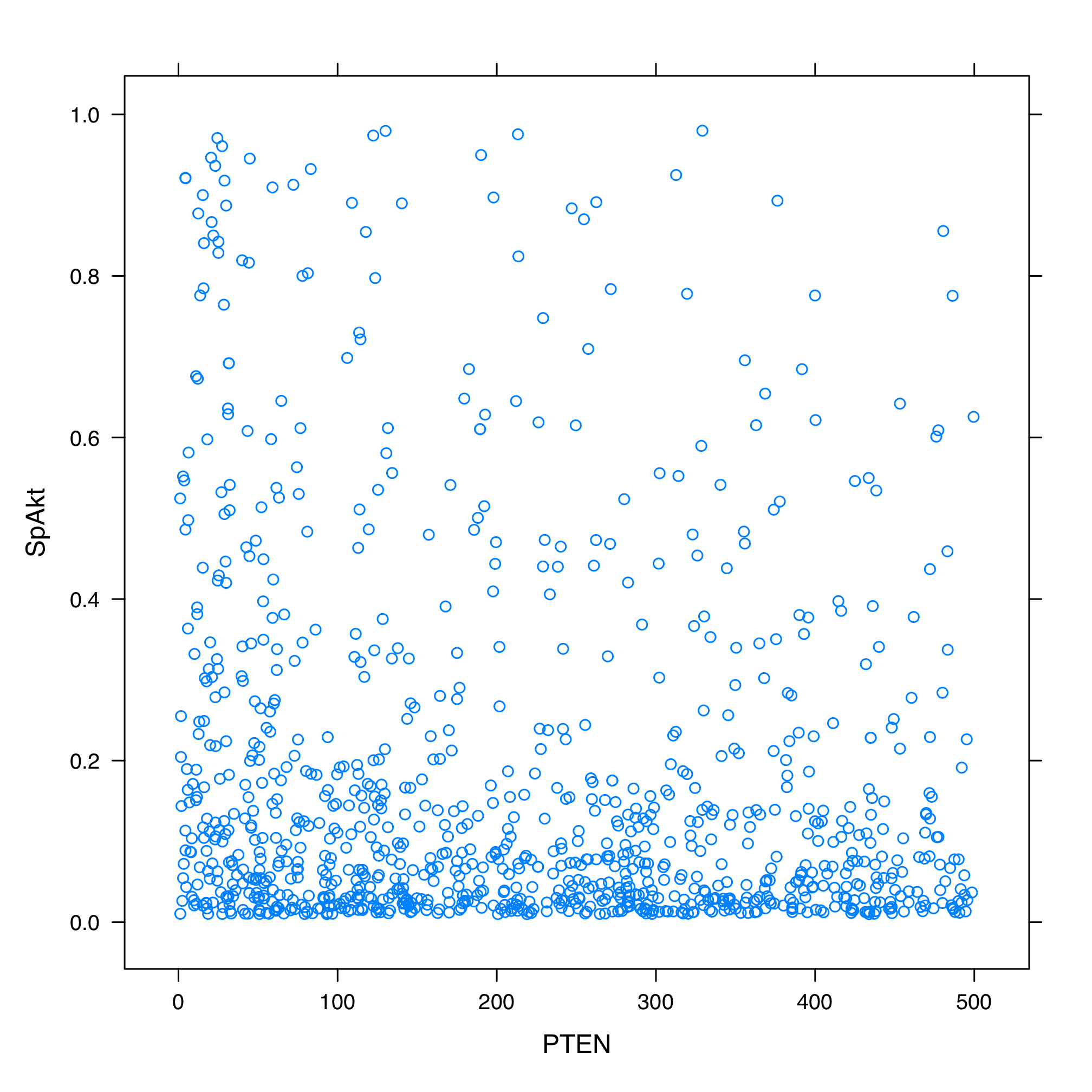


Supplementary Figure S8 (continued)


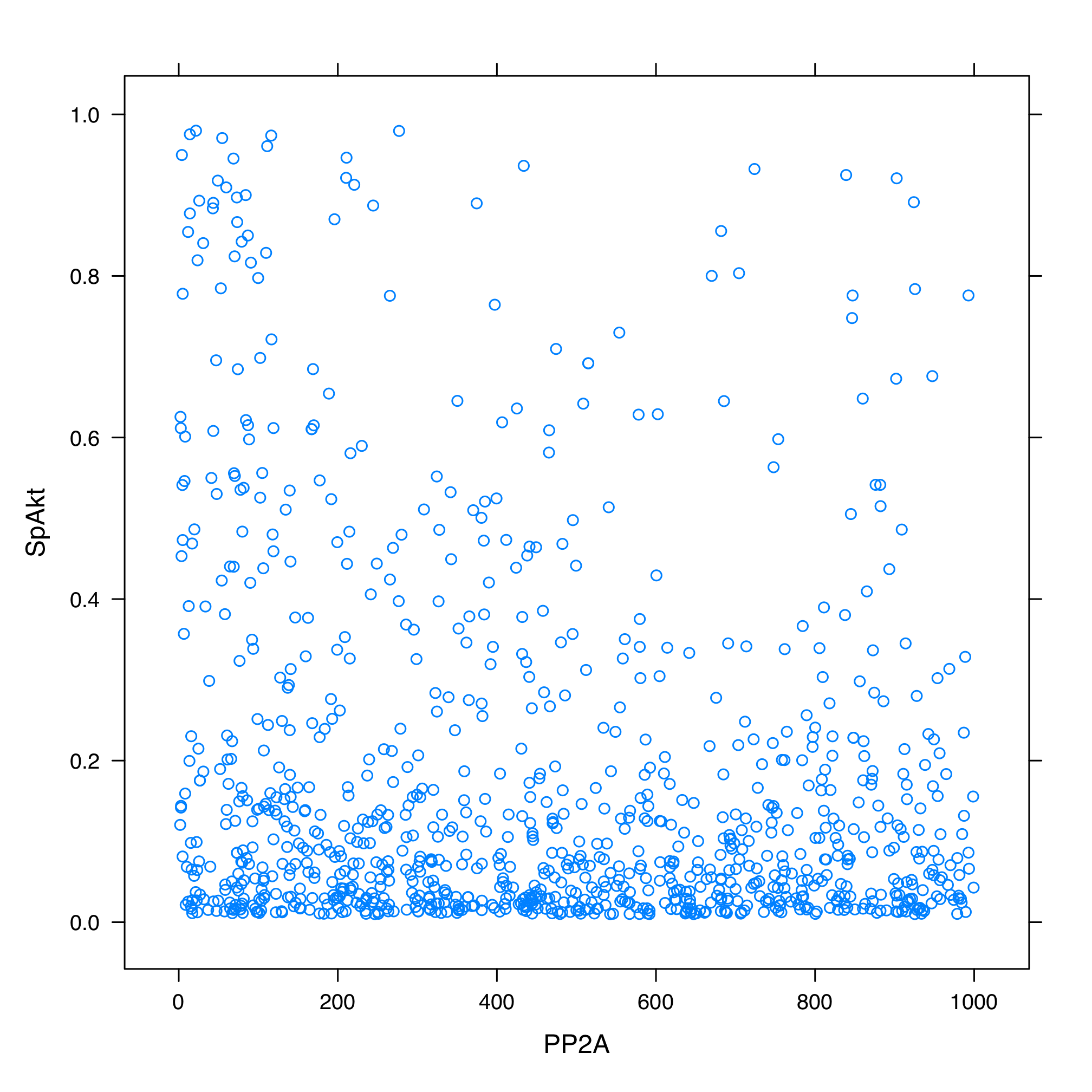

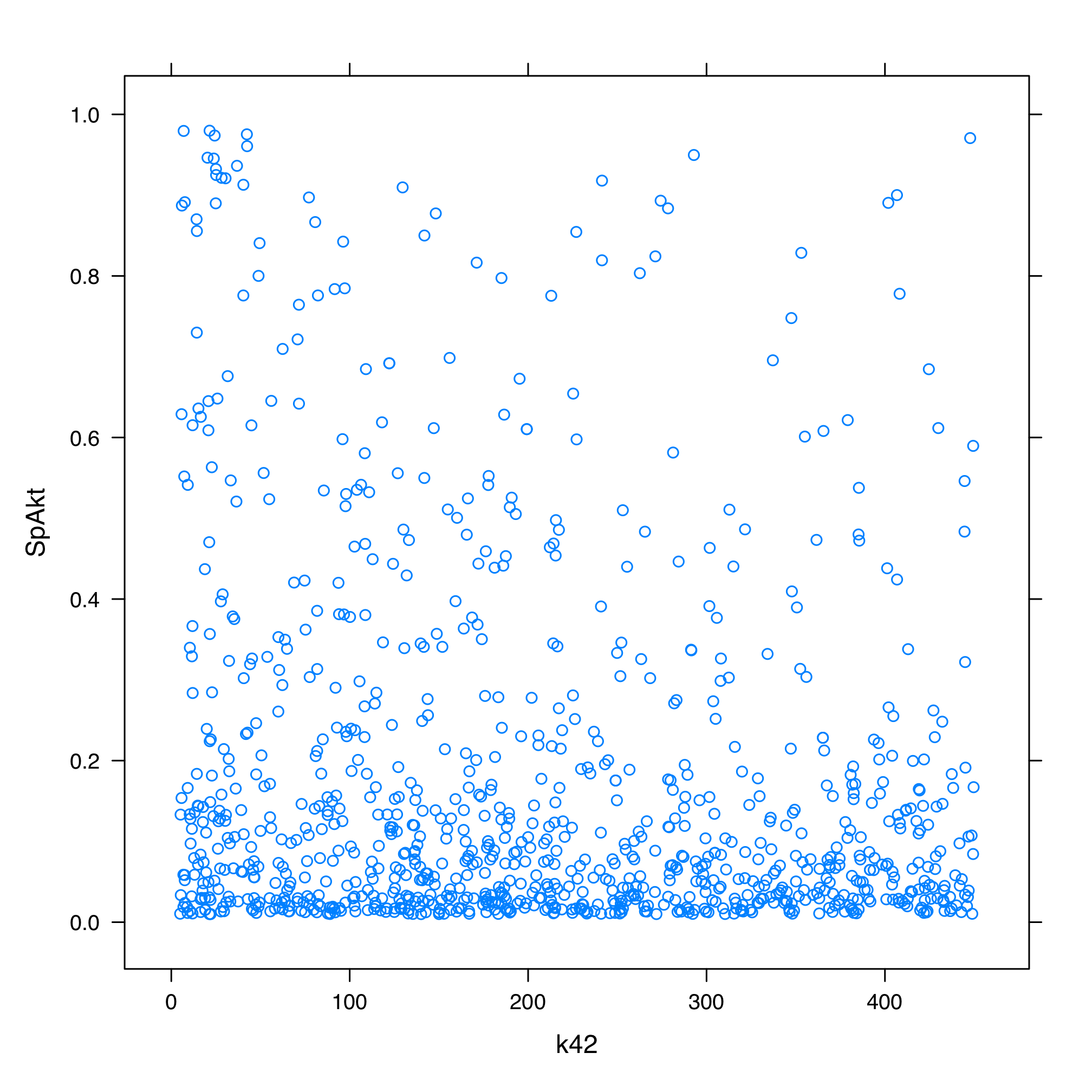


Supplementary Figure S8 (continued)


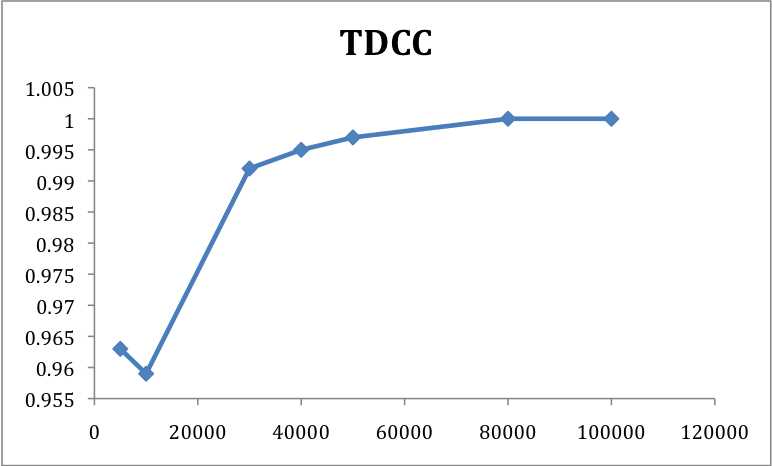


### Supplementary Figure S9. Definition of the optimal sample size N for GSA of ErbB2/3 network model.

TDCC represents the top down coefficient of concordance between parameter rankings obtained in two consecutive sampling experiments, calculated as suggested in [1] and [2]. TDCC values are given on the ordinate, the sample size is shown on the abscissa, so that TDCC(50000) corresponds to TDCC between N=50000 and N=80000; TDCC(80000) is TDCC between N=80000 and N=100000; TDCC(100000) is TDCC between N=100000 and 120000.


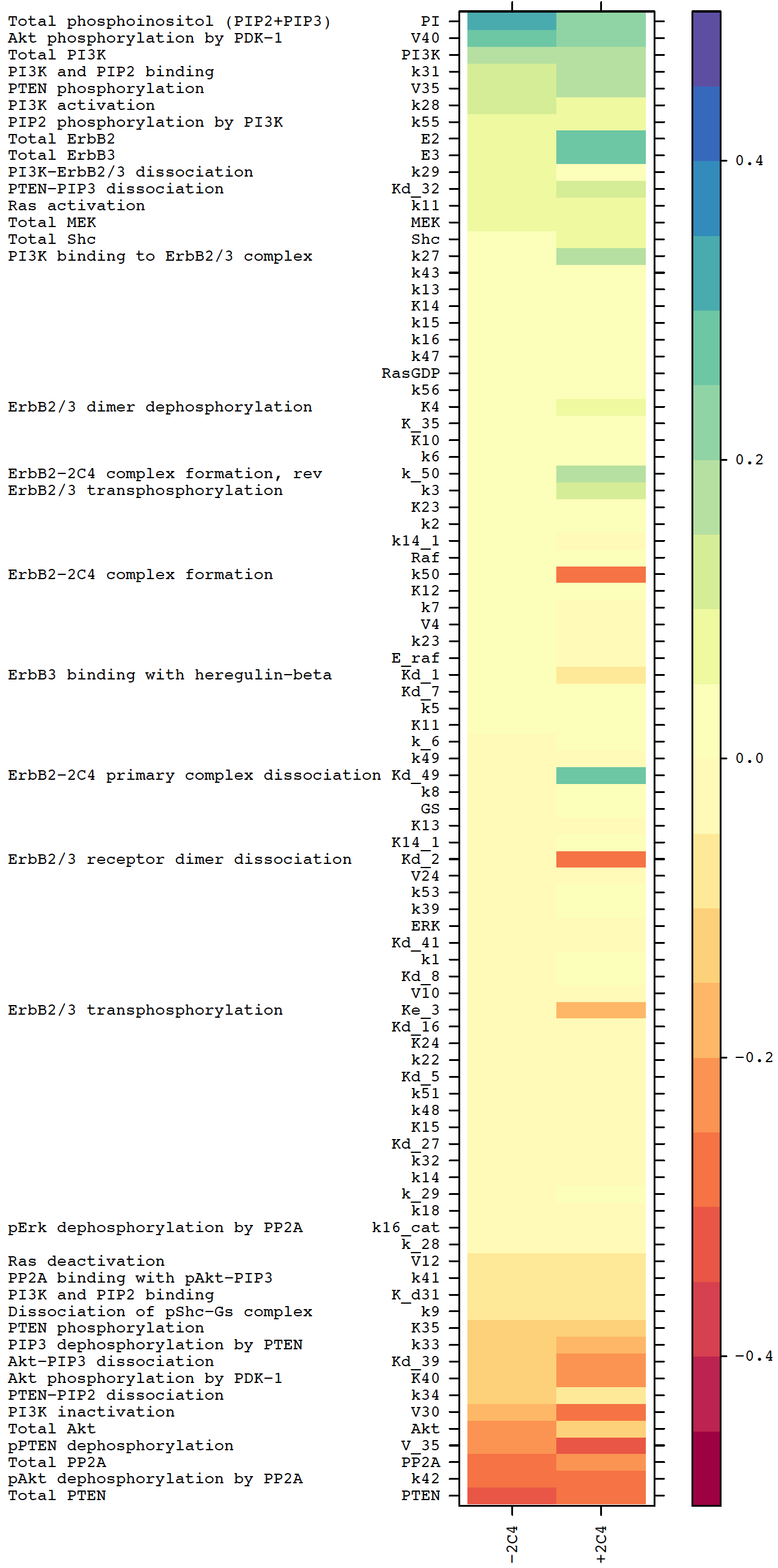


### Supplementary Figure S10. Global sensitivity profile of

Full sensitivity spectrum of integrated pAkt () to the variation of ErbB2/3 network model parameters in the absence (left) and in the presence (right) of pertuzumab (2C4). Comments are provided only for those parameters, whose absolute values of sensitivity indices were higher then 0.05

###

### Analysis of the changes in the sensitivity spectrum of pAkt signal, caused by the introduction of anti-ErbB2 inhibitor pertuzumab.

The sensitivity spectrum of the (Figure 2A of the main manuscript, right column), though retaining most of the sensitivity found for in the absence of the drug, exhibits a number of significant changes.

First of all, introduction of the drug into the ErbB network makes the pAkt signal much more sensitive to the parameters related to the “upstream” part of the signaling pathway, including signal propagation through the level of receptors. In particular, acquires sensitivity to the total concentration of ErbB2 and ErbB3 receptors. High sensitivity of pAkt to ErbB3 has been previously reported in a computational modelling study [3], implicating ErbB3 as the key node in the ErbB network response to ligands that can bind either ErbB3 or EGFR, which led directly to the design of a novel anti-ErbB3 inhibitor MM-121.

Positive correlation of with ErbB2 and ErbB3 also indicates that lower expression levels of these receptors can be associated with a better response to pertuzumab. Indeed, a recent study on the cell line models [4] supported the idea that lower levels of the ErbB3 mRNA are associated with a pertuzumab-sensitive phenotype.

As expected, the analysis revealed high sensitivity of the pAkt signal to the parameters of Pertuzumab-ErbB2 complex formation. In our model, binding of Pertuzumab with ErbB2 was described by a two-stage process – in the first stage (reaction 49) the drug binds with the receptor forming a loosely bound primary complex, which at the next stage (reaction 50) converts into a tightly bound complex. In accordance with this mechanism higher values of the dissociation constant of the primary complex (Kd_49) and the reverse rate constant of tight complex formation (k_50) correlate with higher values of the resulting pAkt output signal. On the contrary, more efficient formation of the tight Per-ErbB2 complex (higher k50) correlates with a lower pAkt signal. This indicates that optimization of the parameters of the drug binding towards a more stable drug-receptor complex could provide more efficient inhibition of the pAkt signal.

In the presence of Pertuzumab the pAkt signal also acquires sensitivity to the parameters of receptor dimerization: it is positively correlated with the rate of ErbB2/ErbB3 transphosphorylation (k3) and the rate of the subsequent PI3K binding to ErbB2/ErbB3 complex (k27), and negatively correlated with Kd_2 (dissociation of the receptor dimer) and K_3 (equilibrium constant of transphosphorylation) – indeed these parameters regulate the efficiency of signal propagation through the receptors, and therefore the more efficient these processes are, and the higher is the rate of PI3K association with the receptor dimer, the higher is the value of the downstream pAkt signal.

Interestingly, in the presence of Pertuzumab the pAkt signal retains high sensitivity to the parameters of PDK-1 (V40 and K40) . This may indicate the potential role of PDK-1 in the emergence of therapeutic resistance to Pertuzumab. In [5] PDK-1 inhibitors are discussed as potential agents to overcome the resistance of breast tumors to tamoxifen, as well as acquired resistance to other anti-cancer treatments.

Similarly, in the presence of the drug the pAkt signal becomes more sensitive (a negative correlation) to the rate of PI3K deactivation (V30) - the process controlling the effective concentration of activated PI3K. This is in agreement with experimental findings implicating PI3K activation mutations in cancer progression and resistance to anti-ErbB drugs [6-8].

It is worth noting that in the presence of Pertuzumab resulting pAkt signal shows somewhat lower sensitivity to the expression level of PTEN, but higher sensitivity to the parameters of the PTEN –phospho-PTEN turnover. Since the rates of PTEN phosphorylation (V35) and dephosphorylation (V_35) control the balance between active and non-active PTEN forms, this indicates that it is the effective concentration of active PTEN, rather than the total PTEN pool, plays a crucial role in regulating the signal propagation through the pAkt branch. Therefore any genetic modification of the enzymes controlling PTEN -pPTEN interconversion may be potentially considered as biomarkers of resistance to ErbB2 inhibitors (E.g. activation mutations in CK2 or Gsk would result in a decrease of active PTEN level and therefore manifest as an effective loss of PTEN activity).


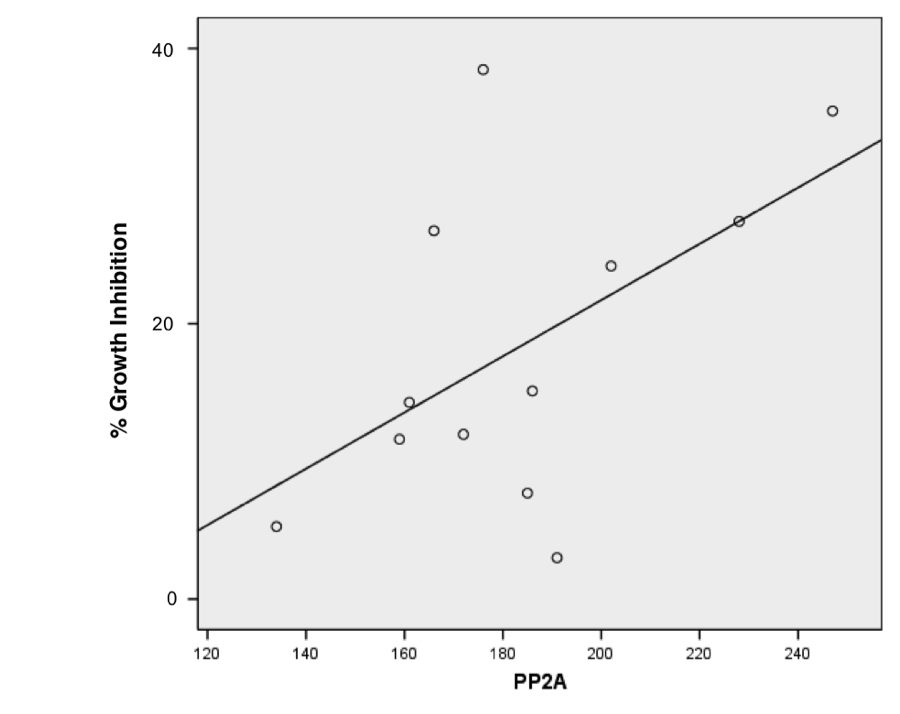


### Supplementary figure S11. Correlation between PP2A expression and cell growth inhibition by pertuzumab

The association of quantitative protein expression of PP2A, measured by reverse phase protein array, on growth inhibition by pertuzumab in a panel of ovarian carcinoma cell lines. There is a moderate positive correlation between PP2A expression and growth inhibition (Spearman’s Rank Correlation 0.434)

### References

1. Iman RL, Conover WJ: **A Measure of Top-down Correlation**. *Technometrics* 1987, **29**(3):351-357.

2. Marino S, Hogue IB, Ray CJ, Kirschner DE: **A methodology for performing global uncertainty and sensitivity analysis in systems biology**. *J Theor Biol* 2008, **254**(1):178-196.

3. Schoeberl B, Pace EA, Fitzgerald JB, Harms BD, Xu L, Nie L, Linggi B, Kalra A, Paragas V, Bukhalid R *et al*: **Therapeutically targeting ErbB3: a key node in ligand-induced activation of the ErbB receptor-PI3K axis**. *Sci Signal* 2009, **2**(77):ra31.

4. Nagumo Y, Faratian D, Mullen P, Harrison DJ, Hasmann M, Langdon SP: **Modulation of HER3 is a marker of dynamic cell signaling in ovarian cancer: implications for pertuzumab sensitivity**. *Mol Cancer Res* 2009, **7**(9):1563-1571.

5. Peifer C, Alessi DR: **New anti-cancer role for PDK1 inhibitors: preventing resistance to tamoxifen**. *Biochem J* 2009, **417**(1):e5-7.

6. Blanco-Aparicio C, Renner O, Leal JF, Carnero A: **PTEN, more than the AKT pathway**. *Carcinogenesis* 2007, **28**(7):1379-1386.

7. Kan Z, Jaiswal BS, Stinson J, Janakiraman V, Bhatt D, Stern HM, Yue P, Haverty PM, Bourgon R, Zheng J *et al*: **Diverse somatic mutation patterns and pathway alterations in human cancers**. *Nature* 2010, **466**(7308):869-873.

8. Coughlin CM, Johnston DS, Strahs A, Burczynski ME, Bacus S, Hill J, Feingold JM, Zacharchuk C, Berkenblit A: **Approaches and limitations of phosphatidylinositol-3-kinase pathway activation status as a predictive biomarker in the clinical development of targeted therapy**. *Breast Cancer Res Treat* 2010.
